# Hydrothermal and Co-Precipitation Combined with Photo-Reduced Preparation of Ag/AgBr/MgBi_2_O_6_ Composites for Visible Light Degradation Toward Organics

**DOI:** 10.3390/nano14231865

**Published:** 2024-11-21

**Authors:** Hsin-Yi Huang, Mudakazhi Kanakkithodi Arun, Sabu Thomas, Mei-Yao Wu, Tsunghsueh Wu, Yang-Wei Lin

**Affiliations:** 1 Department of Chemistry, National Changhua University of Education, 1 Jin-De Road, Changhua City 50007, Taiwan; m0925011@gm.ncue.edu.tw (H.-Y.H.); arunbaburaj99@gmail.com (M.K.A.); 2 School of Nano Science and Nano Technology, Mahatma Gandhi University, Priyadarshini Hills P. O., Kottayam 686560, India; sabuthomas@mgu.ac.in; 3 Department of Physics, Kannur University, Swami Anandatheertha Campus, Payyanur Edat P. O., Kannur 670327, India; 4 School of Post-Baccalaureate Chinese Medicine, China Medical University, 91, Hsueh-Shih Road, Taichung 40424, Taiwan; meiyaowu@mail.cmu.edu.tw; 5 Department of Chemistry, University of Wisconsin-Platteville, 1 University Plaza, Platteville, WI 53818, USA; wut@uwplatt.edu

**Keywords:** photoreduction, AgNPs, AgBr, MgBi_2_O_6_, methylene blue, Z-scheme mechanism

## Abstract

This study developed a MgBi_2_O_6_-based photocatalyst via low-temperature hydrothermal synthesis. AgBr was co-precipitated onto MgBi_2_O_6_, and silver nanoparticles (AgNPs) were photo-reduced onto the surface. The photocatalytic performance, assessed by methylene blue (MB) degradation under white-light LED irradiation (2.5 W, power density = 0.38 W/cm^2^), showed that Ag/AgBr/MgBi_2_O_6_ achieved 98.6% degradation in 40 min, outperforming MgBi_2_O_6_ (37.5%) and AgBr/MgBi_2_O_6_ (85.5%). AgNPs boosted electron-hole separation via surface plasmon resonance, reducing recombination. A Z-scheme photocatalytic mechanism was suggested, where photogenerated carriers transferred across the p–n heterojunction between AgBr and MgBi_2_O_6_, producing reactive oxygen species like superoxide and hydroxyl radicals critical for dye degradation. Thus, the Ag/AgBr/MgBi_2_O_6_ composites possessed excellent photocatalytic performance regarding dyestuff degradation (85.8–99.9% degradation within 40 min) under white-light LED irradiation.

## 1. Introduction

With increased industrialization, significant volumes of organic contaminants, including dyes and waste oils, are discharged into the environment, posing serious threats to biodiversity, human health, and ecosystems [1,2]. Traditional chemical industries contribute heavily to this pollution. Conventional methods for treating contaminants, emerging contaminants of concern (CECs), and wastewater effluents include activated sludge biological systems, activated carbon adsorption, membrane filtration, and chemical treatments. These treatments can be broadly categorized into traditional methods, such as chlorination and biological processes, and advanced oxidation processes (AOPs), including ozonation, ultraviolet irradiation, and potassium permanganate oxidation. AOPs are particularly effective in addressing complex pollutants that are resistant to conventional methods [3,4,5]. To address the diverse advantages and disadvantages of the previously mentioned treatments, greener alternatives such as electrochemical catalysis, semiconductor photocatalysis, Fenton reactions, and supercritical water oxidation have been developed [6,7,8,9,10,11]. These methods aim to overcome specific limitations while offering tailored benefits depending on the nature of the contaminants and treatment goals. However, electrochemical catalysis and supercritical water oxidation face energy and cost limitations, and Fenton-based methods risk secondary pollution from iron sludge [12,13]. In contrast, semiconductor photocatalysis is a renewable and continuous process, offering a promising approach to addressing environmental pollution and energy shortages [14,15].

Bi(III)-based compounds have recently gained attention as photocatalytic materials due to their unique electronic structures. Bismuth compounds and their derivatives are non-toxic and biologically inert, leading to diverse applications [16,17]. Numerous photocatalysts have been synthesized from these compounds, including Bi_2_S_3_, Bi_2_WO_6_, and Bi_2_Ti_2_O_7_ [10,18,19]. Bismuth sulfide and bismuth oxide have energy gaps below 2.0 eV, allowing them to absorb light in the visible spectrum [20,21]. This study focuses on the potential of MgBi_2_O_6_, a noteworthy compound synthesized as an oxide of Bi(V) with a distinctive mirror triple-rutile crystal structure. Unlike Bi(III), where electrons occupy the 6s orbital, Bi(V) in MgBi_2_O_6_ features unfilled 6s orbitals, allowing greater flexibility in electron arrangements and enhanced photocatalytic potential. Although research on magnesium bismuthate is limited, MgBi_2_O_6_ shows promise as a significant contributor to the field of photocatalysis. Its synthesis involves a substitution reaction between magnesium chloride and sodium bismuthate, as follows:

$${{MgCl}_{2}}_{\left(aq\right)}+{2{NaBiO}_{3}}_{\left(aq\right)}\to {{MgBi}_{2}O_{6}}_{\left(s\right)}+{2NaCl}_{\left(aq\right)}$$

with an energy gap of approximately 1.8 eV, MgBi_2_O_6_ facilitates efficient electronic transitions. Literature indicates that MgBi_2_O_6_ exhibits strong visible-light absorption and demonstrates significant activity in degrading methylene blue (MB) under visible light [22,23]. Due to these properties, MgBi_2_O_6_ was selected as the primary photocatalyst in this study, with additional materials incorporated to enhance its degradation performance.

Silver halides (AgX, X = Cl, Br, and I) are susceptible to photo-corrosion in photocatalytic reactions, as Ag(I) is readily reduced to metallic Ag(0) by photogenerated electrons. Silver halides are often anchored to various surfaces to address this limitation, enhancing stability [24,25]. Strategies to mitigate photo-corrosion include coupling silver halides with TiO_2_ via heterojunctions or attaching them to magnetic carriers [26]. Additionally, modifications such as using ZnWO_4_ for band alignment allow photogenerated electrons from AgBr to transfer effectively to ZnWO_4_, thus enhancing stability [27]. Another widely applied method is constructing Z-scheme heterojunctions, which are a type of semiconductor heterojunctions that facilitate efficient charge transfer by utilizing the difference in the energy levels of the two semiconductors, significantly boosting photoactivity and stability [28,29]. Among silver halides, AgCl has strong absorption in the UV range, while AgBr has a visible light absorption edge at 475 nm [30,31,32]. Consequently, this experiment employed AgBr-modified MgBi_2_O_6_ to enhance visible light absorption.

When exposed to visible light, the conduction electrons on the surface of silver nanoparticles (AgNPs) play a crucial role in photocatalysis. They can oscillate collectively at a resonant frequency, a phenomenon known as surface plasmon resonance (SPR) [32,33]. When the frequency of incoming photons aligns with the natural oscillation frequency of surface electrons, plasmonic metal nanostructures (such as gold, silver, and copper) strongly interact with this light, intensifying local SPR effects [33]. This interaction leads to the enhancement of photocatalytic activity within the visible light spectrum [34,35]. Methods for depositing AgNPs onto photocatalytic materials generally involve AgX reduction, which can occur through chemical reduction, thermal reduction, or light-induced reduction. In this experiment, AgBr served as the precursor, and AgNPs were synthesized via light-induced reduction and deposited on AgBr/MgBi_2_O_6_ surfaces. Studies suggest that in Ag/AgX (X = Cl, Br, I) systems, this combination forms a photocatalyst with a narrow energy gap, effectively enhancing visible light responsiveness [36,37]. The Ag/AgBr photocatalyst shows superior catalytic activity compared to Ag/AgCl, as bromine (Br^0^) has a lower electron affinity than chlorine (Cl^0^), resulting in a narrower energy gap (approximately 2.6 eV) [38]. This characteristic improves catalytic efficiency and photostability, although rapid electron-hole recombination still presents challenges for broader applications.

With the rapid advancement of science, technology, and industrial activity, wastewater containing various organic pollutants is increasingly released, contributing significantly to environmental pollution. Since the pioneering work by Fujishima and Honda, TiO_2_ has become widely used as a photocatalyst due to its stability, diversity, and non-toxicity [39,40]. However, TiO_2_‘s application is constrained by its reliance on ultraviolet light for activation [41]. Our study employs pentavalent bismuth-based MgBi_2_O_6_ as a photocatalyst substrate to address this limitation. Although MgBi_2_O_6_ is not the most active among pentavalent bismuth compounds, its effectiveness in degrading organic pollutants like MB can be enhanced by modifying its surface with AgBr, thereby adjusting the band gap for improved photocatalytic performance. In addition, AgNPs are deposited onto the surface of AgBr/MgBi_2_O_6_ via a light-induced reduction method. This process leverages the SPR effect to intensify the local electromagnetic field around the nanoparticles, thereby improving electron-hole separation. Combining AgBr and AgNPs enables enhanced visible light absorption and reduces electron-hole recombination rates. Since SPR is influenced by nanoparticle size, content, and dispersion factors, this study optimizes the light-induced reduction time to control these factors for maximal photodegradation efficiency. Using visible light, the Ag/AgBr/MgBi_2_O_6_ photocatalyst degrades the organic dye (MB), with electrochemical analyses measuring charge separation and electron-hole recombination rates. Additionally, scavenger experiments identify key reactive radicals involved in the photodegradation process. Following the reaction, total organic carbon (TOC) analysis confirms the mineralization of organic pollutants. The prepared Ag/AgBr/MgBi_2_O_6_ photocatalyst shows promise for future applications in sunlight-driven degradation of actual wastewater samples.

## 2. Materials and Methods

### 2.1. Chemicals

All reagents used were obtained from Sigma Aldrich (St. Louis, MO, USA) and were of analytical grade without further purification. Milli-Q ultrapure water was used in all of the experiments.

### 2.2. Characterization of the Prepared MgBi_2_O_6_-Based Composites

An X-ray diffractometer (Model: SMART APEX II, Bruker AXS, Billerica, MA, USA) with Cu Kα radiation (λ = 0.15418 nm) and a scanning electron microscope (SEM) (Model: HITACHI S-4300, Hitachi High Technologies Corporation, Tokyo, Japan) operating at 15 kV and equipped with a QUANTAX Annular XFlash QUAD FQ5060 detector (Bruker Nano, Berlin, Germany) were utilized to determine X-ray diffraction (XRD) patterns and to observe the morphology and composition of MgBi_2_O_6_, AgBr/MgBi_2_O_6_, and Ag/AgBr/MgBi_2_O_6_ composites. Additionally, X-ray photoelectron spectroscopy (XPS) spectra were obtained using a VG ESCA210 electron spectroscope (VG Scientific, West Sussex, UK). For UV–visible diffuse reflectance (UV–Vis DRS) spectra, a UV–visible spectrometer (Model: Evolution 2000, Thermo Fisher Scientific Inc., Madison, WI, USA) with a BaSO_4_ reference was used.

### 2.3. Synthesis of Ag/AgBr/MgBi_2_O_6_ Composites

Detailed synthesis procedures for MgBi_2_O_6_ and AgBr/MgBi_2_O_6_ are provided in the Supplementary Information. To synthesize the Ag/AgBr/MgBi_2_O_6_ composite, weigh 0.5383 g of AgBr/MgBi_2_O_6_ and add it to 30 mL of deionized water to prepare solution A. Stir solution A in a dark room at 600 rpm for 15 min. Weigh 0.5383 g of AgNO_3_, corresponding to the weight ratio of AgNO_3_ to AgBr/MgBi_2_O_6_ of 1:1. Dissolve each amount of AgNO_3_ in 30 mL of deionized water to prepare solution B. Add solution B dropwise to the stirring solution A while illuminating with a 300 W mercury-xenon lamp. Stir the mixture at 600 rpm for 2 h. Allow the mixture to precipitate in a dry and cool place. Wash the precipitated composite three times with deionized water and once with ethanol, followed by centrifugation at 12,000 rpm for 10 min using a high-speed centrifuge after each wash. Transfer the final product into a crucible, cover it with aluminum foil, and dry it in an oven at 60 °C to obtain the Ag/AgBr/MgBi_2_O_6_ composite. Thus, the preparation procedure for the Ag/AgBr/MgBi_2_O_6_ composites in this study was summarized as follows: In a hydrothermal reactor, the synthesis involves a substitution reaction between magnesium chloride and sodium bismuthate to produce MgBi_2_O_6_. AgBr is primarily formed through a co-precipitation reaction between AgNO_3_ and NaBr. Finally, a mercury-xenon lamp is used to irradiate AgNO_3_, facilitating a photo-reduction process that enables silver particles to adhere to the surface of the AgBr/MgBi_2_O_6_ composite.

### 2.4. Photodegradation MB Procedure

MgBi_2_O_6_, AgBr/MgBi_2_O_6_, and Ag/AgBr/MgBi_2_O_6_ composites were tested for MB degradation under low-power white-light LED irradiation. MB was selected as the model molecule due to the following: (a) Common pollutant: MB is a widely used industrial dye and a significant environmental pollutant; (b) Ease of monitoring: Its strong absorption peak at ~665 nm allows easy concentration tracking via UV–Vis spectroscopy; (c) Standard for organic pollutants: MB represents the behavior of many dyes, offering insights into photocatalyst efficiency; (d) Well-documented properties: MB’s known degradation pathways simplify performance evaluation; and (e) Benchmark material: It is commonly used in photocatalytic studies, enabling comparisons with existing research.

An amount of 150 mg of MgBi_2_O_6_, AgBr/MgBi_2_O_6_, or Ag/AgBr/MgBi_2_O_6_ was added to an aqueous MB solution (15 ppm) in a 50 mL quartz flask maintained at 25 °C. Before irradiation, the mixture was stirred in the dark for 30 min to establish adsorption-desorption equilibrium. The suspension was then illuminated with white-light LED lamps (2.5 W, power density = 0.38 W/cm^2^) using a PCX-50C photoreactor (Beijing Perfectlight Technology Co., Ltd., Beijing, China). At set intervals, 1 mL of the suspension was sampled and centrifuged to remove particulate matter. The concentration of MB was measured using a Synergy H1 hybrid multimode microplate reader (Biotek Instruments, Inc., Winooski, VT, USA) at the characteristic absorption wavelength of 665 nm. Total organic carbon (TOC) was measured during degradation using a TOC analyzer (Elementar Acquray, Elementar Analysensysteme GmbH, Langenselbold, Germany). Similar processes were performed for various dyestuffs (methyl red (MR), methyl orange (MO), rhodamine B (RhB), and rhodamine 6G (R6G)).

### 2.5. Evaluation of Charge Separation Efficiency and Recombination Rate

The charge separation efficiency and electron-hole recombination rate of the synthesized composites were assessed following established protocols. Electrochemical impedance spectroscopy (EIS) was performed to measure the charge separation efficiency on the surface of MgBi_2_O_6_, AgBr/MgBi_2_O_6_, and Ag/AgBr/MgBi_2_O_6_ composites after 15 min of white-light LED exposure. A slurry, prepared by mixing 0.1 g of the composite with 1.0 mL of ethanol, was applied onto an indium tin oxide (ITO, 9.42 wt% SnO_2_ and 89.75 wt% In_2_O_3_) substrate (resistivity ~10^−4^ Ω·cm) and then heated at 50 °C for 30 min to ensure complete ethanol evaporation. The coated ITO glass was subsequently annealed at 200 °C for 2 h to stabilize the composite layer and prevent peeling during testing. This prepared sample was analyzed under white-light LED irradiation using a CHI-6122E electrochemical analyzer (CH Instruments Inc., Austin, TX, USA). The electrochemical setup consists of an Ag/AgCl electrode as the reference, a platinum electrode as the auxiliary, and an electrolyte solution composed of 0.1 M KCl and 5.0 mM K_3_[Fe(CN)_6_]. The EIS measurement parameters were configured as follows: applied potential of 1.5 V, amplitude of 0.7 V, high frequency of 1.5 × 10^5^ Hz, and low frequency of 0.03831 Hz. Additionally, the electron-hole recombination rate was determined by capturing luminescence spectra with a photoluminescence (PL) spectrometer (Varian Cary Eclipse, Agilent Technologies, Inc., Santa Clara, CA, USA).

### 2.6. Scavenger Test

To identify the active species involved in the photocatalytic degradation of MB over Ag/AgBr/MgBi_2_O_6_, trapping experiments were performed using ethylenediaminetetraacetic acid disodium salt (EDTA disodium salt) and tert-butanol (t-BuOH) at 1 mmol as scavengers for holes, and hydroxyl radicals, respectively. The experimental setup followed the standard photocatalytic degradation procedure, with a different scavenger added for each test to inhibit the target reactive species selectively. Additionally, MB degradation in an Ag/AgBr/MgBi_2_O_6_ system under nitrogen (N_2_) atmospheres was analyzed to further confirm the role of oxygen radicals as primary reactive species in the degradation pathway.

## 3. Results and Discussion

### 3.1. Characterization

**Figure 1:**
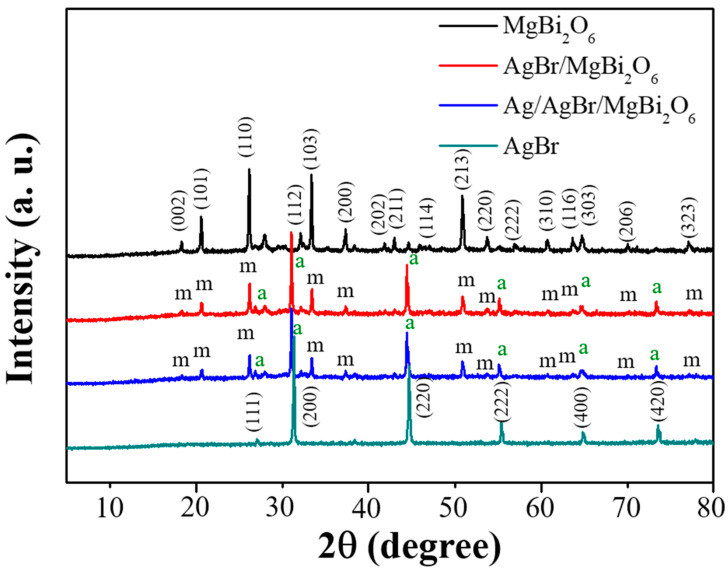
XRD spectra of MgBi_2_O_6_, AgBr/MgBi_2_O_6_, Ag/AgBr/MgBi_2_O_6_, and AgBr. A—AgBr, m—MgBi_2_O_6_.

The XRD patterns of MgBi_2_O_6_, AgBr/MgBi_2_O_6_, Ag/AgBr/MgBi_2_O_6_, and AgBr are presented in Figure 1. The MgBi_2_O_6_ exhibits a mirror-like triple rutile structure, with primary scattering angles at 2θ values of 18.2°, 20.5°, 26.1°, 32.0°, 33.3°, 37.2°, 50.8°, 53.7°, and 64.6°, corresponding to the crystal planes (002), (101), (110), (112), (103), (200), (213), (220), and (303), respectively, in agreement with the JCPDS No. 86-2492 database [42,43,44]. The cubic AgBr structure aligns with JCPDS No. 79-0149, with main scattering angles at 2θ values of 26.7°, 30.9°, 44.3°, 55.0°, 64.5°, and 73.2°, associated with the crystal planes (111), (200), (220), (222), (400), and (420), respectively [45,46]. The figure shows that the synthesized AgBr/MgBi_2_O_6_ and Ag/AgBr/MgBi_2_O_6_ composites exhibit diffraction peaks corresponding to both MgBi_2_O_6_ (m) and AgBr (a), confirming the successful synthesis of the composite materials with crystal planes consistent with the literature standards. In addition, as shown in Figure 1, no other crystal phases can be detected, indicating that no impurities are formed.

As shown in Figure S1, the SEM images reveal that the morphology of MgBi_2_O_6_ consists of cuboidal structures with smooth surfaces, ranging in size from 50 nm to 600 nm, without any attached particles. Figure S2 presents the SEM of AgBr/MgBi_2_O_6_ synthesized via the co-precipitation method, in which AgBr is deposited on the MgBi_2_O_6_ surface. Here, irregular spherical AgBr particles, with sizes between approximately 70 nm and 280 nm, are distributed across the cuboidal MgBi_2_O_6_ structures. Subsequently, Ag/AgBr/MgBi_2_O_6_ was synthesized through the photoreduction of AgNPs, as depicted in Figure S3. In this image, the cuboidal MgBi_2_O_6_ surface is scattered with irregularly shaped AgBr particles, which appear rough and granular at a higher magnification (50.0 K), with Ag ranging from 20 nm to 30 nm in size. EDS-mapping of the photocatalysts indicates a homogeneous distribution of elements throughout each material.

**Figure 2:**
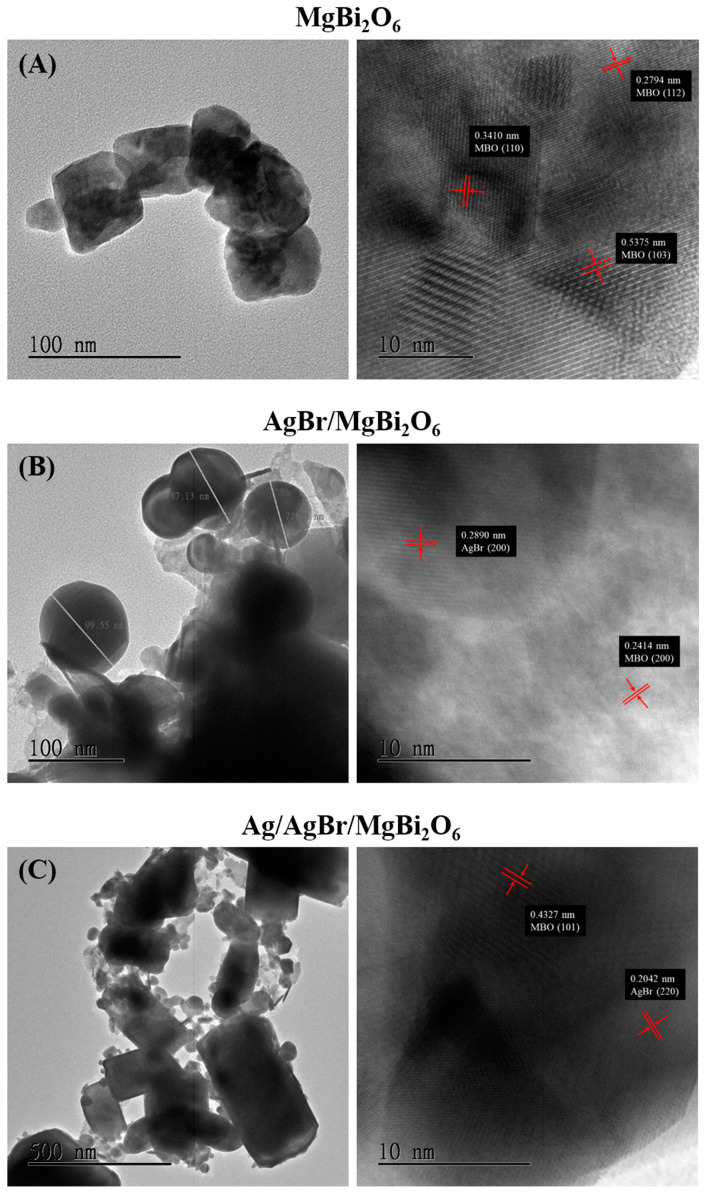
TEM images of (**A**) MgBi_2_O_6_, (**B**) AgBr/MgBi_2_O_6_, and (**C**) Ag/AgBr/MgBi_2_O_6_ composites.

As shown in Figure 2A, MgBi_2_O_6_ displays a square morphology with an average size of approximately 55 nm. This observation is consistent with SEM analysis, which reveals lattice spacing of 0.3410 nm, 0.2794 nm, and 0.5375 nm, corresponding to the MgBi_2_O_6_ crystal planes (110), (112), and (103), respectively. Figure 2B illustrates AgBr/MgBi_2_O_6_ with a morphology similar to that observed in SEM. The AgBr/MgBi_2_O_6_ particle size is around 300 nm, while AgBr measures approximately 85 nm. The lattice spacing of MgBi_2_O_6_ and AgBr are 0.2414 nm and 0.2890 nm, corresponding to the (200) crystal planes of MgBi_2_O_6_ and AgBr, respectively. Figure 2C presents Ag/AgBr/MgBi_2_O_6_, consistent with the SEM observations, where AgBr is attached to the MgBi_2_O_6_ surface, and AgBr’s surface appears rough due to the presence of photoreduced AgNPs of smaller size. At the crystal interface, the lattice spacing of 0.4327 nm and 0.2042 nm are observed, corresponding to the MgBi_2_O_6_ (101) and AgBr (220) crystal planes, respectively.

**Figure 3:**
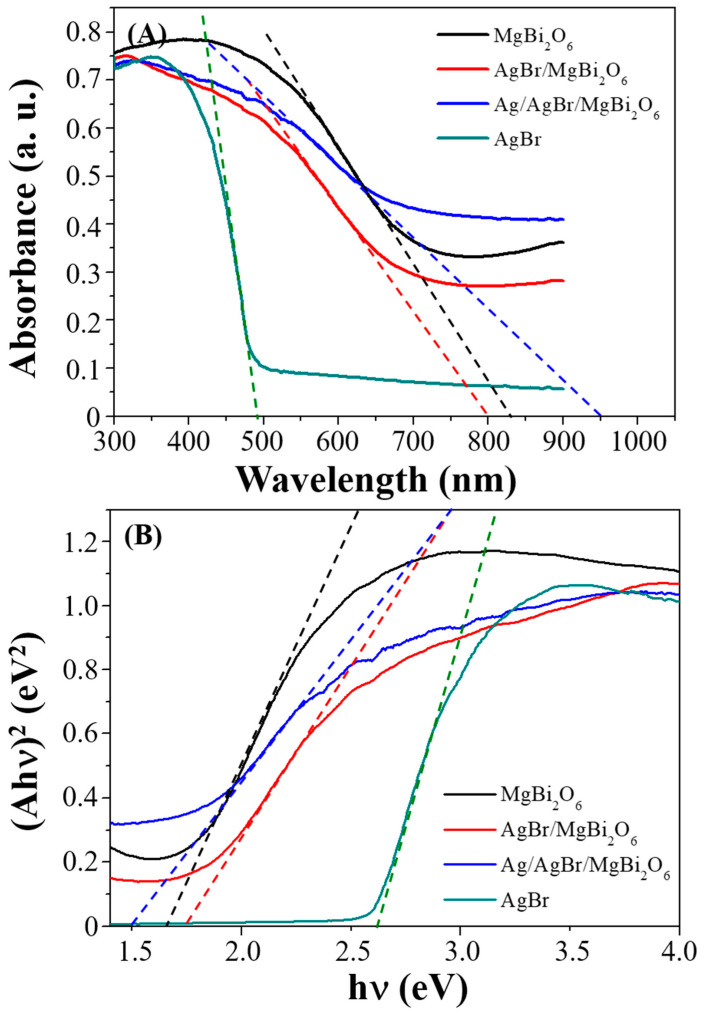
(**A**) UV–Vis DRS spectra and (**B**) the corresponding (Ahν)^2^ versus hν curves of MgBi_2_O_6_, AgBr/MgBi_2_O_6_, Ag/AgBr/MgBi_2_O_6_, and AgBr.

Figure 3A shows the UV–Vis DRS of the photocatalyst materials synthesized in this study. The absorption edges, determined by extrapolating the tangent lines to the *x*-axis, are located at 837 nm, 810 nm, 963 nm, 1029 nm, and 494 nm, corresponding to MgBi_2_O_6_, AgBr/MgBi_2_O_6_, Ag/AgBr/MgBi_2_O_6_, and AgBr, respectively. The band gaps (E_g_) of these semiconductors were calculated using the Kubelka–Munk function and *Tauc* plots, where hν versus (Ahν)^2^ allows for estimating E_g_ from the *x*-axis intercept [22,47,48]. The resulting band gaps for MgBi_2_O_6_, AgBr/MgBi_2_O_6_, Ag/AgBr/MgBi_2_O_6_, and AgBr are 1.66 eV, 1.72 eV, 1.45 eV, and 2.60 eV, respectively (Table 1). E_g_ significantly impacts photocatalytic efficiency and practical applications: materials with higher E_g_ value predominantly absorb ultraviolet light, which constitutes only ~5% of the solar spectrum, thereby limiting their effectiveness under visible light for solar-driven photocatalysis. Conversely, materials with lower E_g_ values can absorb visible light, improving their performance in solar-driven applications such as pollutant degradation and environmental remediation. Furthermore, the band edge positions of MgBi_2_O_6_ and AgBr can be calculated using the following empirical formula:

E_VB_ = χ − E_e_ + 0.5E_g_

E_CB_ = E_VB_ − E_g_

where E_VB_ and E_CB_ are the valance band (VB) edge potential and conduction band (CB) edge potential. χ is the absolute electronegativity, and E_e_ is the potential energy of a free electron in a standard hydrogen electrode (4.5 eV) [44]. The values of χ for MgBi_2_O_6_ and AgBr are 6.11 and 5.81 eV, respectively. Based on the E_g_ of MgBi_2_O_6_ (1.66 eV) and AgBr (2.60 eV), the values of E_CB_ and E_VB_ for MgBi_2_O_6_ are calculated to be 0.78 and 2.44 eV, and those for AgBr are 0.01 and 2.61 eV, respectively.

As shown in Figure 2A, the absorption edge of AgBr at 494 nm falls within the visible light spectrum, indicating that surface modification of AgBr enhances light absorption in this range. Additionally, Figure 2B demonstrates that the E_g_ of Ag/AgBr/MgBi_2_O_6_ is reduced following photoreduction, which lowers the energy barrier for electron transition from the valence to the conduction band and facilitates electron-hole separation.

In this study, the synthesized catalysts were analyzed using an XPS spectrometer to investigate the chemical composition and surface electronic states of the prepared samples. The XPS spectrum of MgBi_2_O_6_, shown in Figure S4, reveals that the sample comprises oxygen, bismuth, and magnesium with atomic ratios of 60.1%, 24.4%, and 15.5%, respectively. The high-resolution spectrum indicates a peak at 1303.2 eV, corresponding to the Mg 1s level of Mg(II). After peak deconvolution, the binding energies of 49.57 eV and 48.35 eV are assigned to Mg(II) and Mg, respectively, with Mg(II) representing approximately 69.3% of the total Mg content. In the high-resolution Bi spectrum, two distinct peaks at 163.8 eV and 163.3 eV correspond to Bi 4f_5/2_, while peaks at 158.6 eV and 158.0 eV correspond to Bi 4f_7/2_, indicative of Bi(V) and Bi(III) states [49,50]. The high-resolution O spectrum shows peaks at 531.1 eV, 530.7 eV, and 529.0 eV, attributed to O, O^−^, and O^2−^, respectively.

The XPS spectrum of AgBr/MgBi_2_O_6_ is presented in Figure S5, showing that the sample comprises O, Bi, Ag, Mg, and Br with atomic proportions of 55.8%, 20.7%, 10.7%, 6.8%, and 6.1%, respectively. In the high-resolution XPS spectrum of Ag, two main peaks are observed for Ag 3d_3/2_ and Ag 3d_5/2_. Deconvolution of these peaks reveals binding energies at 373.4 eV and 367.4 eV for Ag(I) and at 373.1 eV and 367.2 eV for metallic Ag, with Ag(I) being the predominant form. The high-resolution Br spectrum displays peaks at 68.68 eV and 67.65 eV, corresponding to Br 3d_3/2_ and Br 3d_5/2_, respectively. The Mg XPS spectrum shows a peak at 1303.2 eV, attributed to the Mg 1s orbital of Mg(II), consistent with the spectrum in Figure S4. Further deconvolution identifies peaks at 49.59 eV and 48.65 eV, corresponding to Mg(II) and metallic Mg, respectively. In the high-resolution Bi spectrum, Bi 4f_5/2_ and Bi 4f_7/2_ peaks are split into four peaks, with 163.7 eV and 158.5 eV assigned to Bi(V) and 163.5 eV and 158.1 eV to Bi(III) [49,50]. For oxygen, the high-resolution XPS reveals peaks at 531.1 eV, 530.8 eV, and 529.1 eV, corresponding to O, O^−^, and O^2−^, with O^2−^ being the dominant form at approximately 44.3% [51].

The XPS spectrum of Ag/AgBr/MgBi_2_O_6_ is presented in Figure S6, showing that the sample contains O, Ag, Bi, Br, and Mg, with atomic proportions of 44.4%, 22.4%, 15.4%, 12.1%, and 5.7%, respectively. After photo-reduction, the increased silver content confirms the successful formation of silver nanoparticles on the surface. Consequently, the atomic ratio of Ag to Br in Ag/AgBr/MgBi_2_O_6_ rose to 1.85 compared to AgBr/MgBi_2_O_6_ (the atomic ratio of Ag to Br is 1.67). The high-resolution XPS spectrum of Ag displays characteristic peaks at Ag 3d_3/2_ and Ag 3d_5/2_. After peak deconvolution, four distinct peaks are observed, with binding energies of 373.7 eV and 368.0 eV corresponding to Ag(I), and 373.6 eV and 367.6 eV corresponding to metallic Ag. The high-resolution Br spectrum shows peaks at 69.18 eV and 68.12 eV, attributed to Br 3d_3/2_ and Br 3d_5/2_, respectively. For Mg, high-resolution XPS peaks at 49.6 eV and 48.0 eV correspond to Mg(II) and Mg, with Mg(II) accounting for approximately 56.4% of the Mg present. In the Bi spectrum, the Bi 4f_5/2_ and Bi 4f_7/2_ peaks are split into four peaks: 164.0 eV and 158.7 eV for Bi(V), the predominant oxidation state, and 163.5 eV and 158.3 eV for Bi(III) [49,50]. The high-resolution O spectrum shows peaks at 532.1 eV, 530.8 eV, and 529.3 eV, corresponding to O, O^−^, and O^2−^, with O^2−^ as the dominant form at approximately 42.7% [51].

The resulting binding energies for the prepared MgBi_2_O_6_, AgBr/MgBi_2_O_6_, and Ag/AgBr/MgBi_2_O_6_ are also close to the previously reported values [22,44,52,53].

### 3.2. Degradation Performance

Prior to evaluating photocatalytic degradation, the optimized preparation conditions for Ag/AgBr/MgBi_2_O_6_, including the precursor mixing process (ultrasound or cell disruption), the light source used during photo-degrading MB (UV or white-light LED), the weight ratios of AgNO_3_ to AgBr/MgBi_2_O_6_, and the photo-reduction reaction time, were systematically examined. Detailed results and discussions of these optimization factors are provided in the Supplementary Information (Figure S7).

**Figure 4:**
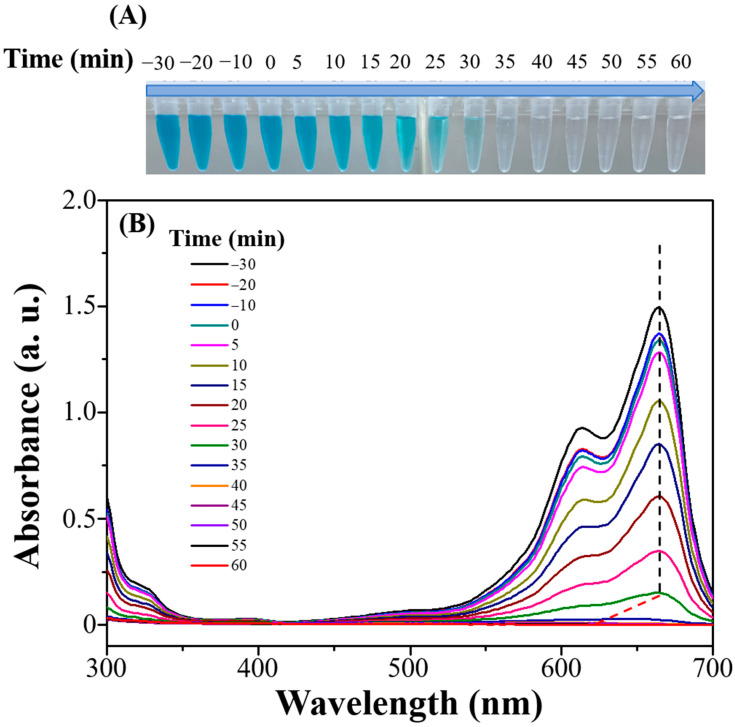
(**A**) Photograph images and (**B**) UV–Vis spectra of MB solutions under visible light irradiation using Ag/AgBr/MgBi_2_O_6_ composites at different times.

The Ag/AgBr/MgBi_2_O_6_ synthesized under optimized conditions effectively degraded 15 ppm of MB. UV–Vis spectra of MB solutions at different times are illustrated in Figure 4B. The maximum absorption peak of MB is observed at 665 nm, with absorbance values progressively decreasing over the degradation period. Literature suggests that the degradation process involves two primary mechanisms: decolorization and mineralization [54,55]. Decolorization refers to the breakdown of chromophores and the fragmentation of organic molecules into smaller ones, often resulting in peak shifts or the appearance of new peaks. In contrast, mineralization directly converts organic molecules into CO_2_ and H_2_O, maintaining the same peak wavelength without new peak formation. Based on the results in Figure 4B, Ag/AgBr/MgBi_2_O_6_ was utilized for the degradation of MB. During the first 30 min of illumination (indicated by the black dashed line), mineralization was identified as the predominant degradation mechanism. After 30 min, a shift in the maximum absorption peak (red dashed line) was observed, indicating a transition toward decolorization. These spectral changes align with the gradual alteration in dye color over time, as illustrated in Figure 4A.

**Figure 5:**
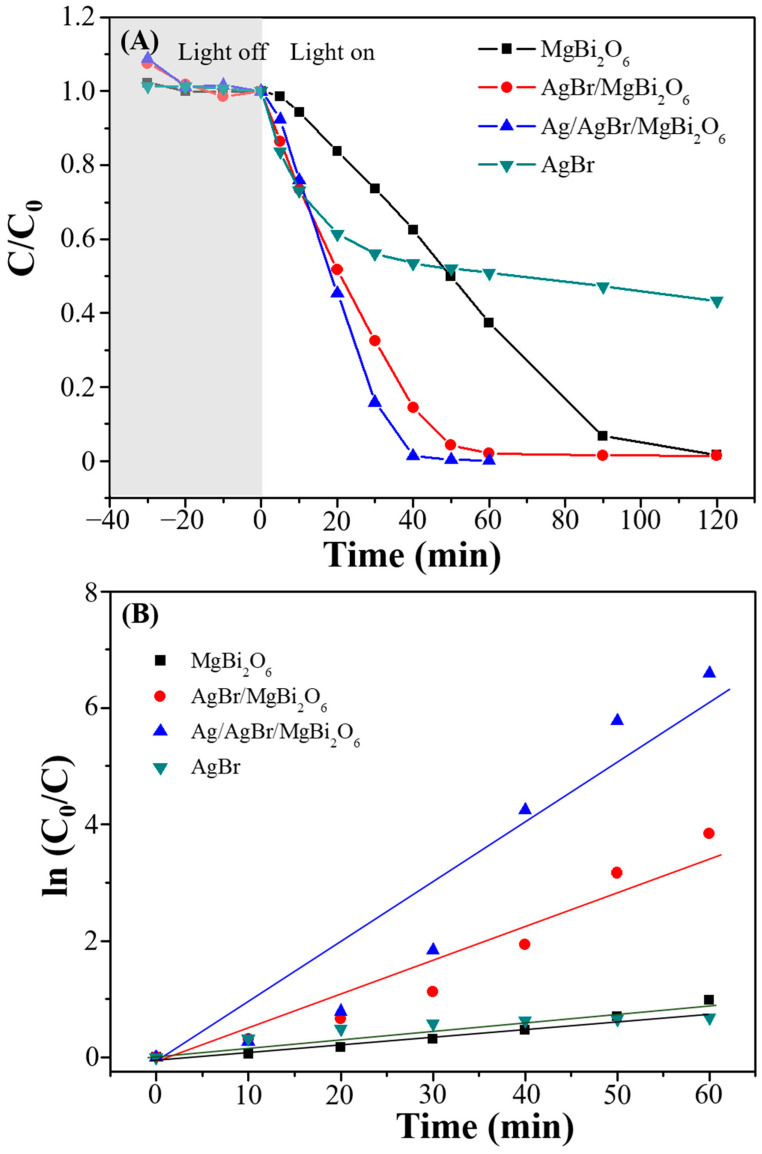
(**A**) Degradation performance of MB and (**B**) pseudo-first-order reaction for MB degradation by using MgBi_2_O_6_, AgBr/MgBi_2_O_6_, and Ag/AgBr/MgBi_2_O_6_ composites.

The photocatalytic performance of the synthesized catalysts (MgBi_2_O_6_, AgBr/MgBi_2_O_6_, Ag/AgBr/MgBi_2_O_6_, and AgBr) was assessed for MB degradation under visible light irradiation. Figure 5A illustrates the changes in MB concentration (C/C_0_) over irradiation time for each photocatalyst, where C_0_ is the initial MB concentration, and C is the concentration at time t. MB demonstrates negligible degradation without a photocatalyst under visible light irradiation (We will present the results later). The results in Figure 5A reveal significant differences in MB degradation across the photocatalysts within a 120 min observation period, with Ag/AgBr/MgBi_2_O_6_ emerging as the most effective catalyst. In addition, based on the light-off adsorption experiments (30 min) shown in Figure 5A, the observed differences in saturation coverage highlight a potential increase in surface area or improved adsorption properties for AgBr/MgBi_2_O_6_ and Ag/AgBr/MgBi_2_O_6_ composites. This approach could serve as a preliminary indicator of effective surface area changes for different catalysts. The kinetics of MB degradation were further analyzed (Figure 5B), indicating a pseudo-first-order reaction. From the data in Figure 5B, the pseudo-first-order rate constants (k) for MB degradation by MgBi_2_O_6_, AgBr/MgBi_2_O_6_, Ag/AgBr/MgBi_2_O_6_, and AgBr were determined to be 0.0162, 0.0661, 0.1223, and 0.0102 min^−1^, respectively. Notably, the rate constant for Ag/AgBr/MgBi_2_O_6_ is approximately 7.55 times greater than that of MgBi_2_O_6_. The pseudo-first-order kinetic equation, rate constants, and correlation coefficients are summarized in Table 2.

### 3.3. Degradation Mechanism

**Figure 6:**
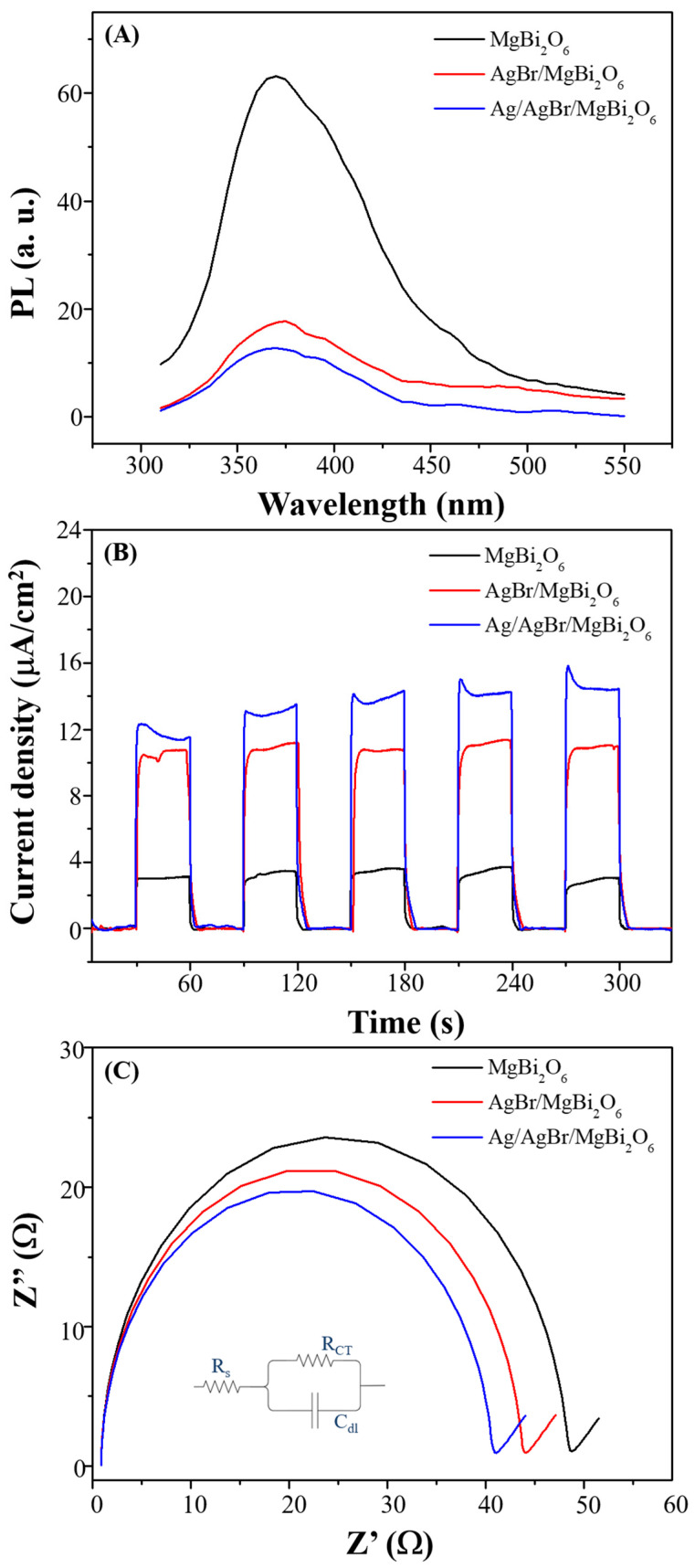
(**A**) PL, (**B**) Current density, and (**C**) EIS analysis of MgBi_2_O_6_, AgBr/MgBi_2_O_6_, and Ag/AgBr/MgBi_2_O_6_ composites.

The photocatalytic reaction depends on the effective separation of photogenerated electron-hole pairs. In the PL spectrum, emitted light results from electron transitions from higher to lower energy states, reflecting free electron recombination. Consequently, PL intensity is proportional to the likelihood of electron-hole pair recombination. To assess whether the synthesized photocatalyst tends to facilitate electron transition from the conduction to the valence band post-excitation, which would reduce its lifespan, we measured the PL intensity of the powder samples at an excitation wavelength of 325 nm and an emission wavelength of 400 nm, as shown in Figure 6A. Compared to MgBi_2_O_6_, the PL intensity of AgBr/MgBi_2_O_6_ and Ag/AgBr/MgBi_2_O_6_ was significantly lower, suggesting the formation of an n-type electron and p-type hole space charge region upon light excitation. Additionally, the internal electric field established at the p–n heterojunction enhances charge separation and accelerates charge transfer. The p–n heterojunction formation suppresses electron-hole recombination, thereby enhancing the photocatalytic efficiency. This demonstrates that p–n heterojunction materials such as AgBr/MgBi_2_O_6_ and Ag/AgBr/MgBi_2_O_6_ effectively limit electron-hole recombination, improving photocatalytic performance.

The prepared MgBi_2_O_6_, AgBr/MgBi_2_O_6_, and Ag/AgBr/MgBi_2_O_6_ photocatalysts were individually coated onto the surface of an ITO electrode to fabricate a custom photocurrent electrode, enabling measurement of current density (μA/cm^2^). The photocurrent measurement reflects the combined effects of photogenerated electron quantity and electron-hole separation efficiency. The highest photocurrent intensity observed in the heterojunction indicates an increased number of photogenerated electrons and efficient charge transfer, signifying a higher electron-hole pair separation efficiency. As shown in Figure 6B, the photocurrent response is reproducible over multiple cycles of visible light switching, confirming the synthesized photocatalyst’s stability under illumination. Among the samples, Ag/AgBr/MgBi_2_O_6_ exhibited the highest current density, approximately 14 ± 2 μA/cm^2^, followed by AgBr/MgBi_2_O_6_ (10 ± 0.2 μA/cm^2^) and MgBi_2_O_6_ (3.5 ± 0.5 μA/cm^2^). Notably, the photocurrent trends also correlate with degradation efficacy, reinforcing the impact of post-illumination current response on photocatalytic performance.

To investigate the charge transfer properties of the photocatalyst, EIS was employed. Equivalent circuit modeling, as shown in the inset of Figure 6C, identifies R_s_ as the internal resistance, C_dl_ as the electric double-layer capacitance, and R_CT_ as the charge transfer resistance. In the Nyquist plots, a smaller semicircle radius corresponds to a lower R_CT_ value, indicating enhanced charge transfer efficiency and more effective separation of photogenerated electron-hole pairs. The R_CT_ values for MgBi_2_O_6_, AgBr/MgBi_2_O_6_, and Ag/AgBr/MgBi_2_O_6_ are 47.20 Ω, 42.58 Ω, and 39.54 Ω, respectively. Among the photocatalysts, Ag/AgBr/MgBi_2_O_6_ exhibits the lowest R_CT_ value, reflecting the highest charge transfer efficiency. This also aligns with its narrower bandgap, facilitating effective electron-hole pair separation and thus improving photocatalytic performance.

**Figure 7:**
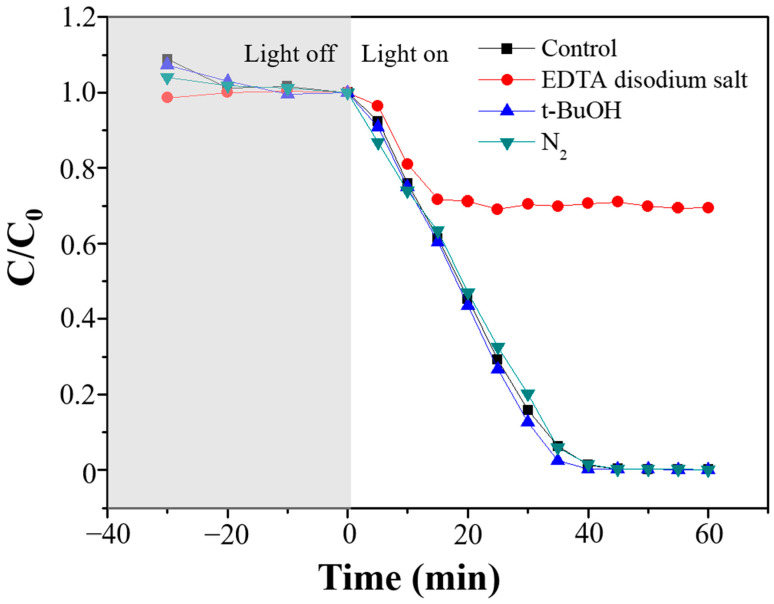
Scavenger tests of Ag/AgBr/MgBi_2_O_6_ composites.

To identify the main reactive species involved in the MB degradation process, a series of trapping experiments were conducted using specific scavengers. EDTA disodium salt (1 mmol) was added to capture holes (h^+^), while t-BuOH (1 mmol) was used to scavenge hydroxyl radicals (•OH). Additionally, nitrogen gas was bubbled into the solution for 15 min before and continuously during the degradation experiment to remove dissolved oxygen, thereby inhibiting the formation of superoxide radicals (•O_2_^−^). The effects of these scavengers on MB degradation were assessed by comparing the degradation efficiency in their presence to a control experiment without any scavenger. A significant reduction in MB degradation efficiency upon adding a scavenger indicates the involvement of the corresponding reactive species in the photocatalytic process. As shown in Figure 7, adding EDTA disodium salt reduced the MB degradation efficiency to 29.4% after 40 min, demonstrating that holes (h^+^) are the main reactive species responsible for MB degradation by the Ag/AgBr/MgBi_2_O_6_ photocatalyst.

**Scheme 1:**
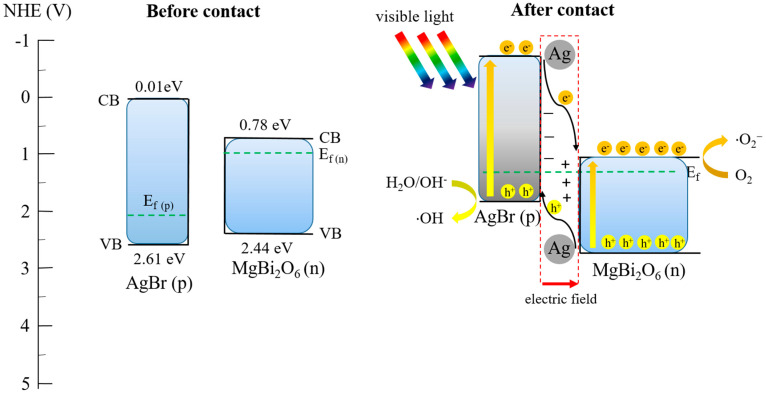
Possible mechanism of photodegradation using Ag/AgBr/MgBi_2_O_6_ composites.

Based on the above discussion, a proposed mechanism for the photocatalytic reaction is illustrated in Scheme 1. AgBr, a p-type semiconductor, and MgBi_2_O_6_, an n-type semiconductor, form a p–n heterojunction, establishing an internal electric field that accumulates negative charges on AgBr and positive charges on MgBi_2_O_6_ [22,44]. Under visible light excitation, AgBr strongly absorbs light energy, promoting electrons to its conduction band. These electrons are then transferred to AgNPs due to the internal electric field. Enhanced by the SPR effect of AgNPs, the electrons are further transferred to the conduction band of MgBi_2_O_6_, where they react with oxygen to form •O_2_^−^ radicals. Simultaneously, holes in the valence band of MgBi_2_O_6_ migrate to the valence band of AgBr, generating •OH radicals upon reacting with water. This efficient electron and hole separation, enhanced by the SPR effect of AgNPs, significantly reduces electron-hole recombination. The generated electrons, holes, and reactive species, such as •O_2_^−^ and •OH, facilitate the degradation of MB through processes like ring opening, demethylation, and molecular decomposition. Alternatively, due to its high oxidation potential, MB can undergo direct redox reactions, yielding non-toxic byproducts, including CO_2_ and H_2_O.

### 3.4. Applications

**Figure 8:**
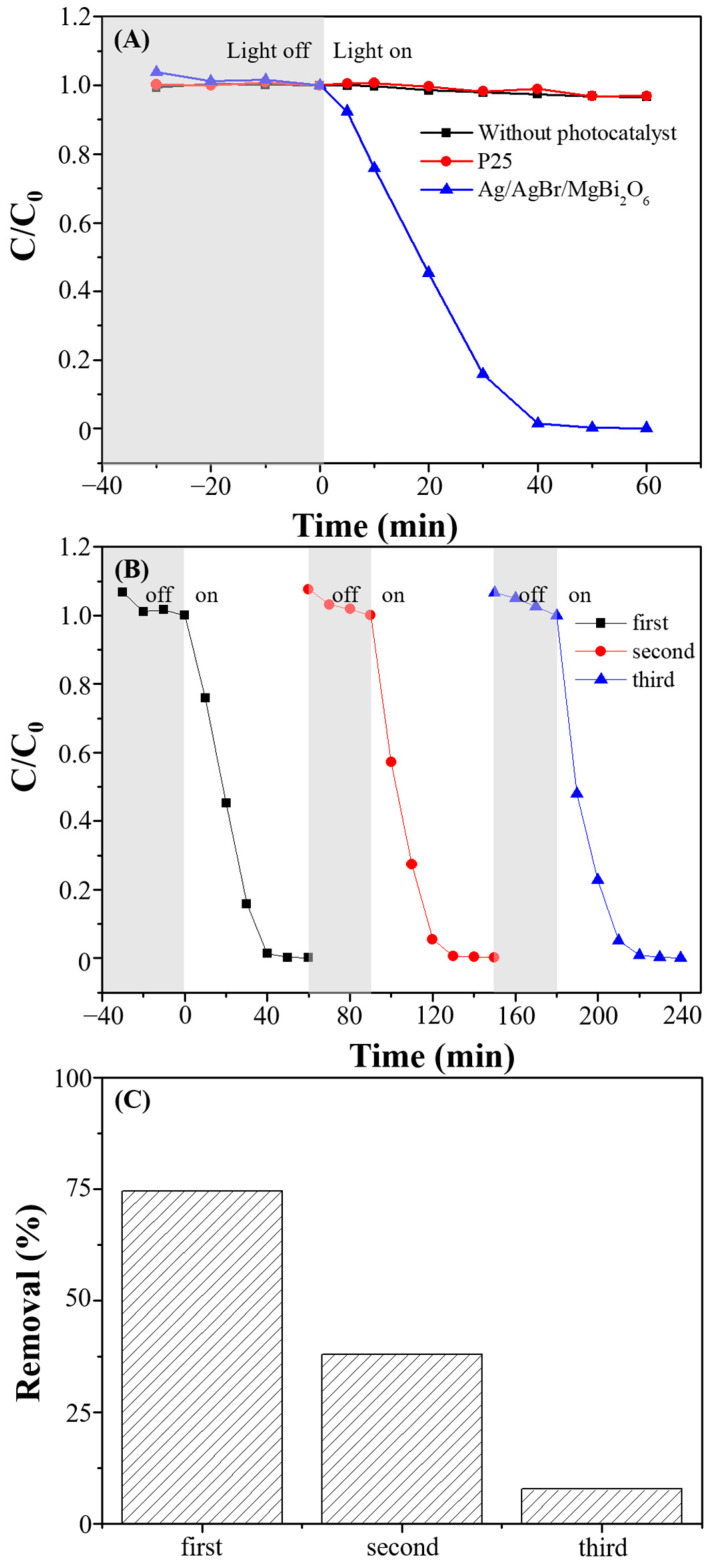
(**A**) Degradation performances of MB in the absence of catalyst and presence of P25 and Ag/AgBr/MgBi_2_O_6_ composites, (**B**) recycling used abilities, and (**C**) TOC experiments of Ag/AgBr/MgBi_2_O_6_ composites.

As shown in Figure 8A, MB demonstrates negligible degradation without a photocatalyst. When compared with the commercially available photocatalyst P25, the degradation efficiency of MB reached only 1.8% after 40 min of reaction. Although P25 offers advantages such as high stability, various forms, and non-toxicity, its effectiveness is limited by its band gap (~3.2 eV), which restricts practical applications under visible light. In this study, the Ag/AgBr/MgBi_2_O_6_ photocatalyst was successfully synthesized, achieving effective electron-hole pair separation upon visible light excitation, leading to significant degradation efficiency.

A photocatalyst facilitates the photodegradation process by interacting with reactants during the reaction without being consumed, maintaining its mass and chemical properties unchanged. This study employs heterogeneous catalysis, which is advantageous due to its recyclability and alignment with principles of green chemistry and environmental sustainability for practical applications. Degradation tests were repeated after drying and reusing the material to assess the photocatalyst’s reusability. As illustrated in Figure 8B, after three consecutive cycles, the photocatalyst demonstrated high degradation efficiency (1st used: 98.6%, 2nd used: 99.3%, and 3rd used: 99.1% within 40 min), indicating that Ag/AgBr/MgBi_2_O_6_ possesses excellent stability and ease of recovery in solution. Notably, an increase in dye adsorption over cycles suggests potential changes in the material’s surface properties. Despite this, Ag/AgBr/MgBi_2_O_6_ consistently proved to be an effective and recyclable photocatalyst.

Total carbon (TC) encompasses both TOC and total inorganic carbon (TIC). When the visible color of degraded organic pollutants is fully removed, TOC analysis helps determine whether the process involves decolorization or complete mineralization. TOC measurement techniques include direct measurement and differential methods [56]. The direct measurement involves acidifying the sample and purging it with nitrogen to remove carbon dioxide from carbonate reactions, then measuring the remaining TOC. The differential method involves sequential injection into a high-temperature and a low-temperature furnace. The high-temperature furnace oxidizes all carbon (TC), while the low-temperature furnace measures only TIC. Subtracting TIC from TC yields the TOC value.

This experiment employed the direct measurement, wherein samples were acidified with phosphoric acid and purged with high-purity nitrogen. TOC was then measured following UV oxidation with peroxypyrosulfate and detected via infrared analysis. Using 15 ppm MB as the target pollutant, the TOC reduction after photodegradation is illustrated in Figure 8C, showing a 74.6% removal rate after the first use. This result confirms that full conversion of MB into CO_2_ and H_2_O was not achieved. This discrepancy arises because MB degradation involves a combination of pathways, including mineralization and partial breakdown into intermediate organic compounds (Figure 4B). Mineralization plays a significant role in the early stages, but the persistent intermediates limit complete TOC removal. In addition, the photocatalytic efficiency remained high across subsequent cycles, and the TOC exhibited significant reduction trends, likely indicating that decolorization dominated after the first used cycle. Moving forward, we will employ advanced analytical techniques such as liquid chromatography-mass spectrometry to identify and quantify the intermediates formed during MB degradation. This will provide a more comprehensive understanding of the photocatalytic mechanism and the nature of residual organic compounds.

**Figure 9:**
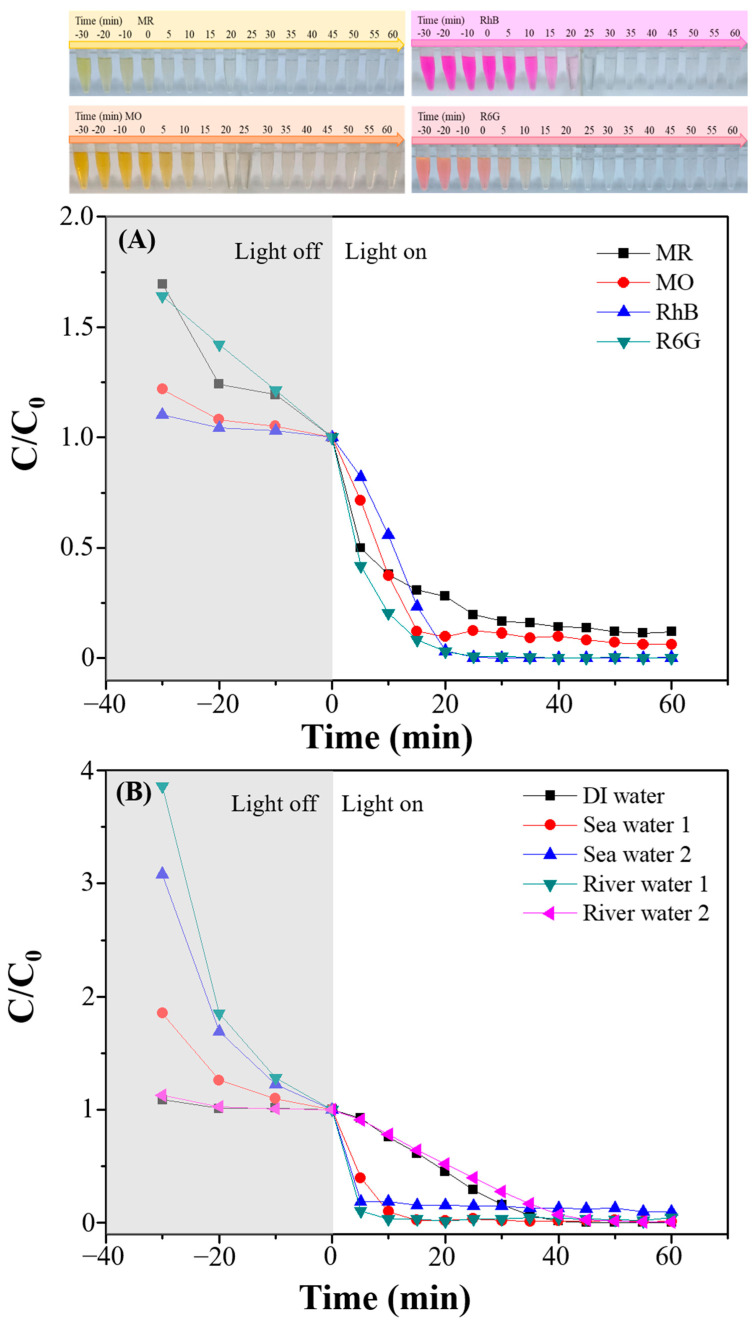
(**A**) Degradation performances of different dyestuff and photograph images of different dyestuff solutions, (**B**) degradation MB in the different environmental water samples using Ag/AgBr/MgBi_2_O_6_ composites.

In addition to MB, MR, MO, RhB, and R6G were selected as target dyes for degradation under identical experimental conditions. Each dye’s peak absorbance was monitored per unit time, as illustrated in Figure 9A. The degradation efficiencies after 40 min were as follows: MR at 85.8%, MO at 90.1%, RhB at 99.9%, and R6G at 99.8%. While some dye was removed upon reaching adsorption-desorption equilibrium in dark conditions, indicating Ag/AgBr/MgBi_2_O_6′_s adsorption effect, the initial degradation might involve decolorization rather than full mineralization. RhB exhibited minimal adsorption issues, achieving a 96.7% degradation efficiency within just 20 min. These findings demonstrate that the Ag/AgBr/MgBi_2_O_6_ photocatalyst synthesized in this study shows strong catalytic activity for diverse dyes, as evidenced by the color changes of MR, MO, RhB, and R6G solutions, as shown in the top photograph of Figure 9A.

In real water samples, such as river and seawater, bacteria, heavy metals, minerals, and other substances can influence photodegradation reactions. To evaluate the material’s feasibility in practical applications and assess whether these factors reduce the photocatalytic efficiency of Ag/AgBr/MgBi_2_O_6_, we compared real water samples with deionized water. The water samples included coastal seawater from Taichung City, Taiwan (Seawater 1), Changhua County, Taiwan (Seawater 2), river water from Tainan City, Taiwan (River Water 1), and from Taichung City, Taiwan (River Water 2). After pre-treatment, each sample was spiked with 15 ppm MB as the target pollutant. The degradation efficiency for each water source is shown in Figure 9B, reaching 96.0%, 92.6%, 87.2%, and 98.5% within 40 min, respectively. Notably, the degradation efficiency in Taichung River Water was closest to that observed in deionized water, while other samples exhibited strong dye adsorption. This difference may be attributed to mineral interference in seawater or the presence of porous substances that adsorb and decolorize the dye.

Table 3 compares the degradation performances using similar photocatalysts. The Ag/AgBr/MgBi_2_O_6_ photocatalyst, with its unique combination of AgNPs and AgBr with MgBi_2_O_6_, demonstrates significant promise for environmental remediation. Its ability to function efficiently under visible light conditions makes it suitable for real-world applications where UV light sources may not be practical. The high stability and reusability of the photocatalyst contribute to its economic viability, reducing the need for frequent replacement and minimizing environmental impact.

The innovative integration of AgNPs and AgBr with MgBi_2_O_6_ not only enhances the photocatalytic efficiency but also broadens the scope of photocatalytic applications. This material can potentially be applied to degrade a wide range of dyestuff, making it a versatile solution for pollution control. In summary, the Ag/AgBr/MgBi_2_O_6_ photocatalyst represents a significant advancement in photocatalysis, combining high efficiency, stability, and versatility. Its development paves the way for more effective and sustainable approaches to environmental cleanup and offers a robust platform for future research and innovation in photocatalytic materials.

The potential toxicity of the photocatalyst was not explicitly assessed in this study. However, previous research on similar photocatalysts has demonstrated that toxicity is largely dependent on the leaching of heavy metals or reactive intermediates during the photocatalytic process. To mitigate such concerns, the materials used in this study, such as MgBi_2_O_6_, AgBr, and Ag nanoparticles, were designed to be stable under photocatalytic conditions. Leaching studies could be conducted in future work to ensure minimal release of harmful ions, such as Ag^+^, into treated water. In future investigations, cytotoxicity assays or environmental toxicity assessments, such as testing on aquatic organisms or evaluating the photocatalyst’s impact on microbial communities, will be performed to comprehensively evaluate its safety for environmental applications.

## 4. Conclusions

This study successfully developed a novel MgBi_2_O_6_ photocatalyst modified with AgBr and AgNPs through hydrothermal synthesis, co-precipitation, and photo-reduction methods. The Ag/AgBr/MgBi_2_O_6_ composite exhibited remarkable photocatalytic performance, achieving 98.6% degradation of MB within 40 min under 2.5 W white-light LED irradiation. This efficiency far surpassed the performance of MgBi_2_O_6_ and AgBr/MgBi_2_O_6_, underscoring the superior activity of the composite. The enhanced performance of Ag/AgBr/MgBi_2_O_6_ is attributed to its Z-scheme photocatalytic mechanism, which facilitates efficient electron-hole separation and minimizes recombination losses. The formation of a p–n heterojunction between AgBr and MgBi_2_O_6_ generates an internal electric field, enabling directional charge transfer and exclusive pathways for photogenerated carriers. Additionally, the SPR effect of Ag nanoparticles significantly boosts visible light absorption, further enhancing photocatalytic activity. The composite demonstrated excellent stability and reusability, retaining its degradation efficiency over multiple cycles. Its capability to degrade various dyes and in real water samples highlights its versatility and potential for real-world environmental remediation applications. These findings position Ag/AgBr/MgBi_2_O_6_ as a promising candidate for sustainable pollutant degradation under visible light irradiation.

## Figures and Tables

**Table 1:** Eg, CB, and VB of MgBi_2_O_6_, AgBr/MgBi_2_O_6_, Ag/AgBr/MgBi_2_O_6_, Ag/MgBi_2_O_6_, and AgBr.

Series	Eg (eV)	CB (eV)	VB (eV)
MgBi_2_O_6_	1.66	0.78	2.44
AgBr/MgBi_2_O_6_	1.72	-	-
Ag/AgBr/MgBi_2_O_6_	1.45	-	-
AgBr	2.60	0.01	2.61

**Table 2:** Kinetic parameters for the pseudo-first-order reaction equation of MgBi_2_O_6_, AgBr/MgBi_2_O_6_, Ag/AgBr/MgBi_2_O_6_, Ag/MgBi_2_O_6_, and AgBr.

Series	k (min^−1^)	Pseudo-First-Order Reaction	R^2^
MgBi_2_O_6_	0.0162	y = 0.0162x − 0.1002	0.9509
AgBr/MgBi_2_O_6_	0.0661	y = 0.0661x − 0.4071	0.9473
Ag/AgBr/MgBi_2_O_6_	0.1223	y = 0.1223x − 0.8789	0.9405
AgBr	0.0102	y = 0.0102x + 0.1725	0.8068

**Table 3:** Comparisons of degradation performances using similar catalysts.

Series	Preparation	Degradation Performance	Real Samples	Ref.
AgBr/MgBi_2_O_6_	Hydrothermal, coprecipitation	0.2 g catalyst/10 ppm MB/94% degradation in 35 min (300 W Xe lamp)	-	[44]
AgX/MgBi_2_O_6_ (X = Cl, Br, I)	Co-precipitation	0.15 g catalyst/15 ppm MB/99.5% degradation in 20 min (2.5 W white-light LED irradiation)	-	[22]
Ag/MgBi_2_O_6_	Heat treatment	0.2 g catalyst/10 ppm MB/98.6% degradation in 120 min (300 W Xe lamp)	-	[43]
AgBr-BiOBr	Co-precipitation	0.1 g catalyst/20 ppm RhB/80% degradation in 10 min (300 W Xe lamp)	-	[57]
AgBr-Ag-BiOBr	Precipitation	15 mg catalyst/15 ppm RhB/97% degradation in 20 min (300 W Hg lamp)	-	[58]
Ag-AgBr/ZnWO_4_	Hydrothermal, precipitation, sonication	2 g catalyst/50 ppm AR18/91% degradation in 60 min (500 W Xe lamp)	-	[27]
Ag/AgBr/Bi_2_CrO_6_	Microwave-assisted hydrothermal, in-situ precipitation, photo-reduction method	0.1 g catalyst/10 ppm RhB/98.0% degradation in 30 min (250 W Xe lamp)	-	[34]
Ag/AgBr/MgBi_2_O_6_	Hydrothermal, co-precipitation, photo-reduction	0.15 g catalyst/15 ppm MB/98.6% degradation in 40 min (2.5 W white-light LED irradiation)	Sea and river water	This study

## Data Availability

Data is contained within the article.

## Supplementary Materials

The following supporting information can be downloaded at: https://www.mdpi.com/article/10.3390/nano14231865/s1, Experiment section: synthesis of MgBi_2_O_6_, AgBr/MgBi_2_O_6_, and Ag/AgBr/MgBi_2_O_6_ under different conditions. Results and discussion: Optimum conditions for the synthesis of Ag/AgBr/MgBi_2_O_6_. Figure S1: SEM/EDS-mapping of MgBi_2_O_6_; Figure S2: SEM/EDS-mapping of AgBr/MgBi_2_O_6_ composites; Figure S3: SEM/EDS-mapping of Ag/AgBr/MgBi_2_O_6_ composites; Figure S4: XPS analysis of MgBi_2_O_6_; Figure S5: XPS analysis of AgBr/MgBi_2_O_6_; Figure S6: XPS analysis of Ag/AgBr/MgBi_2_O_6_; Figure S7: Optimum conditions of Ag/AgBr/MgBi_2_O_6_ composites.
