# Supplementary material for: Hydrothermal and Co-Precipitation Combined with Photo-Reduced Preparation of Ag/AgBr/MgBi2O6 Composites for Visible Light Degradation Toward Organics
Source: Nanomaterials (Basel). 2024 Nov 21;14(23):1865. doi: 10.3390/nano14231865 (PMC11643669; doi:10.3390/nano14231865)
Supplement: Supplementary file 1 [file nanomaterials-14-01865-s001.zip › nanomaterials-3324400-supplementary.pdf]

Supporting information

Hydrothermal and Co-Precipitation Combined with Photo-Reduced Preparation of $Ag/AgBr/MgBi_2O_6$ Composites for the Visible-Light Degradation toward Organics

Hsin-Yi Huang[1], Mudakazhi Kanakkithodi Arun[1, 2, 3], Sabu Thomas[2], Mei-Yao Wu[4], Tsunghsueh Wu[5], and Yang-Wei Lin[1,*]

[1] Department of Chemistry, National Changhua University of Education, 1 Jin-De Road, Changhua City 50007, Taiwan

[2] School of Nano Science and Nano Technology, Mahatma Gandhi University, Priyadarshini Hills P. O., Kottayam 686560, India.

[3] Department of Physics, Kannur University, Swami Anandatheertha Campus, Payyanur Edat P. O., Kannur 670327, India

[4] School of Post-baccalaureate Chinese Medicine, China Medical University, 91, Hsueh-Shih Road, Taichung 40424, Taiwan

[5] Department of Chemistry, University of Wisconsin-Platteville, 1 University Plaza, Platteville, Wisconsin 53818-3099, USA

* Correspondence: Department of Chemistry, National Changhua University of Education, Changhua City 50007, Taiwan; Tel: +886-4-7211190; E-mail: linywjerry@cc.ncue.edu.tw (Y.-W. Lin)

Experiment section

Synthesis of $MgBi_2O_6$ using a cell disruptor or under ultrasonic irradiation

To synthesize $MgBi_2O_6$, it dissolves 1.40 g of $NaBiO_3$ in 30 mL of deionized water to form solution A. Separately, it dissolves 2.03 g of $MgCl_2 \cdot 6H_2O$ in 30 mL of deionized water to create solution B. It combines solutions A and B, and homogenizes the mixture using a cell disruptor or ultrasonic irradiation for 30 minutes. It transfers the mixture into a Teflon-lined autoclave and conducts a low-temperature hydrothermal synthesis at 130 °C for 6 hours. Then, it washes the resulting sample three times with deionized water and once with ethanol, followed by centrifugation at 12,000 rpm for 10 minutes after each washing step. Finally, it dries the collected material in an oven at 60 °C to obtain $MgBi_2O_6$.

Synthesis of $AgBr/MgBi_2O_6$

To prepare the $AgBr/MgBi_2O_6$ composite, dissolve 0.31 g of NaBr in 30 mL of deionized water to create solution A. Add 0.5383 g (0.001 mol) of $MgBi_2O_6$ to solution A and stir at 600 rpm for 15 minutes to achieve uniform dispersion. Separately, dissolve 0.51 g of $AgNO_3$ in 30 mL of deionized water to prepare solution B. Add solution B dropwise to solution A under constant stirring in a dark room at 600 rpm for 2 hours. Allow the resulting mixture to precipitate in a cool, dry environment. Wash the precipitated photocatalyst three times with deionized water and once with ethanol, centrifuging at 12,000 rpm for 10 minutes after each wash. Finally, dry the material in an oven at 60 °C to yield the $AgBr/MgBi_2O_6$ composite.

Synthesis of $Ag/AgBr/MgBi_2O_6$ under different conditions

To fabricate the $Ag/AgBr/MgBi_2O_6$ composite, disperse 0.5383 g of $AgBr/MgBi_2O_6$ in 30 mL of deionized water to form solution A. Stir solution A in a dark room at 600 rpm

for 15 minutes. Prepare solution B by dissolving various amounts of $AgNO_3$ (0.1794 g, 0.2692 g, 0.5383 g, 1.0766 g, and 1.6149 g) in 30 mL of deionized water, corresponding to weight ratios of $AgNO_3$ to $AgBr/MgBi_2O_6$ of 0.33:1, 0.5:1, 1:1, 2:1, and 3:1, respectively. Add solution B dropwise to solution A while illuminating the mixture with a 300 W mercury-xenon lamp. Stir the mixture at 600 rpm for reaction times of 1, 2, or 4 hours to produce different samples. Allow the precipitated composite to settle in a cool, dry place. Wash the material three times with deionized water and once with ethanol, followed by centrifugation at 12,000 rpm for 10 minutes after each washing step. Transfer the final product to a crucible, cover with aluminum foil, and dry in an oven at 60 °C to obtain the $Ag/AgBr/MgBi_2O_6$ composite.

Results and discussion

Optimum conditions for the synthesis of $Ag/AgBr/MgBi_2O_6$

Based on methods from previous literature, $MgBi_2O_6$ was synthesized by treating $NaBiO_3$ and $MgCl_2·6H_2O$ with ultrasonic irradiation for 30 minutes. For $AgBr/MgBi_2O_6$ synthesis, the mixture was stirred at 600 rpm for 2 hours to ensure even distribution of AgBr and $MgBi_2O_6$. To investigate the effect of ultrasonic-assisted synthesis on the photocatalytic degradation properties, an ultrasonic reactor or cell disruptor was used, and photocatalytic degradation experiments were performed to assess the catalytic efficiency of photocatalysts produced by different methods. The synthesized composites are coded as follows, S1: $NaBiO_3$ and $MgCl_2·6H_2O$ were subjected to ultrasound for 30 minutes to produce $MgBi_2O_6$, followed by reaction with AgBr at 600 rpm for 2 hours. S2: $NaBiO_3$ and $MgCl_2·6H_2O$ were sonicated for 30 minutes to synthesize $MgBi_2O_6$, then AgBr and $MgBi_2O_6$ were combined in a cell disruptor for 2 hours. S3: $NaBiO_3$ and $MgCl_2·6H_2O$ were reacted in a cell disruptor for 30 minutes to form $MgBi_2O_6$, followed by stirring with AgBr at 600 rpm for 2 hours.

S4: $NaBiO_3$ and $MgCl_2\cdot 6H_2O$ were reacted in a cell disruptor for 30 minutes to produce $MgBi_2O_6$, and then AgBr and $MgBi_2O_6$ were further reacted in a cell disruptor for 2 hours. The photocatalysts S1-S4 were evaluated for their photocatalytic properties using an LED multi-channel photocatalytic reaction device with an intensity of 0.38 W/cm^2. MB served as the target pollutant for degradation assessment.

As shown in Figure S7A, the photocatalysts S1, S2, S3, and S4 degraded 90.5%, 71.6%, 98.0%, and 73.5% of MB, respectively, within 60 minutes, with S3 demonstrating the highest photodegradation efficiency. The photocatalytic results indicate that the S2 and S4, synthesized with a cell disruptor during AgBr modification, exhibited relatively lower photocatalytic performance. This may be due to the instability of AgBr, where the high-energy cell disruptor potentially damages AgBr or reduces silver ions when reacting with $MgBi_2O_6$. Based on these findings, S3 was confirmed as the optimal method for synthesizing AgBr/$MgBi_2O_6$, and this synthesis approach was used in subsequent experiments.

The surface of AgBr/$MgBi_2O_6$ was modified with photo-reduced AgNPs to synthesize Ag/AgBr/$MgBi_2O_6$. This study investigated the photocatalytic performance under two light sources: white-light LED lamps (visible light irradiation) and ultraviolet light (UV irradiation). As shown in Figure S7B, the degradation rates reached 99.6% and 84.3% after 60 minutes of irradiation, respectively. The high efficiency under visible light is attributed to the absorption edge of AgBr at 494 nm, which falls within the visible spectrum, promoting the dispersion of Ag ions across the AgBr surface and facilitating the formation of AgNPs. The synthesized Ag/AgBr/$MgBi_2O_6$ demonstrated enhanced visible light absorption and energy uptake, further improving photocatalytic performance. Conversely, for composite materials synthesized under UV light irradiation, it is speculated that UV exposure may compromise the structural integrity

of $AgBr/MgBi_2O_6$, leading to a reduction in photodegradation efficiency (84.3%) compared to $AgBr/MgBi_2O_6$ synthesized without UV irradiation (98.0%).

After confirming the use of visible light to photo-reduce AgNPs, different amounts of $AgNO_3$ solid power were added and irradiated under visible light for 2 hours to synthesize $Ag/AgBr/MgBi_2O_6$ and evaluate its photocatalytic effect, as shown in Figure S7C. Here, m_0 refers to the photoreduction synthesis of $AgBr/MgBi_2O_6$ without additional $AgNO_3$, while m_1, m_2, m_3, m_4, and m_5 represent weight ratios of $AgNO_3$ to $AgBr/MgBi_2O_6$ of 0.33:1, 0.5:1, 1:1, 2:1, and 3:1, respectively, with $AgBr/MgBi_2O_6$ fixed at 0.5383 g. Photocatalytic degradation was assessed over 40 minutes, with degradation rates for m_0, m_1, m_2, m_3, m_4, and m_5 at 65.2%, 99.2%, 92.3%, 99.6%, 99.4%, and 94.6%, respectively. In a 30-minute reaction, m_0 to m_5 showed rates of 52.3%, 94.5%, 94.4%, 97.1%, 91.9%, and 75.7%. The optimal photocatalytic efficiency was observed with m_3, where $AgNO_3$ and $AgBr/MgBi_2O_6$ were mixed in a 1:1 weight ratio and reduced under visible light. Different weight ratios of $AgNO_3$ to $AgBr/MgBi_2O_6$ led to different degradation efficiency, likely for two reasons: (1) AgNPs produced from different $AgNO_3$ amounts may vary in size, affecting the SRP and thus the material's light absorption; (2) an excess of AgNPs on the $AgBr/MgBi_2O_6$ surface may block active catalytic sites, reducing interaction with target pollutants and impairing the catalytic effect.

After standardizing the light source and weight ratio of $AgNO_3$ to $AgBr/MgBi_2O_6$, the impact of photoreduction time on the photocatalytic activity of the composite was investigated, as illustrated in Figure S7D. Following photo-reduction for 1, 2, and 4 hours under visible light irradiation and a 40-minute degradation test, the photocatalytic efficiencies were 78.1%, 99.6%, and 94.7%, respectively. Variations in photo-reduction time lead to different degrees of AgNP formation, influencing nanoparticle size and the

extent of SPR. Experimental results indicate that a 2-hour photo-reduction period yields $Ag/AgBr/MgBi_2O_6$ with the optimal photocatalytic performance.

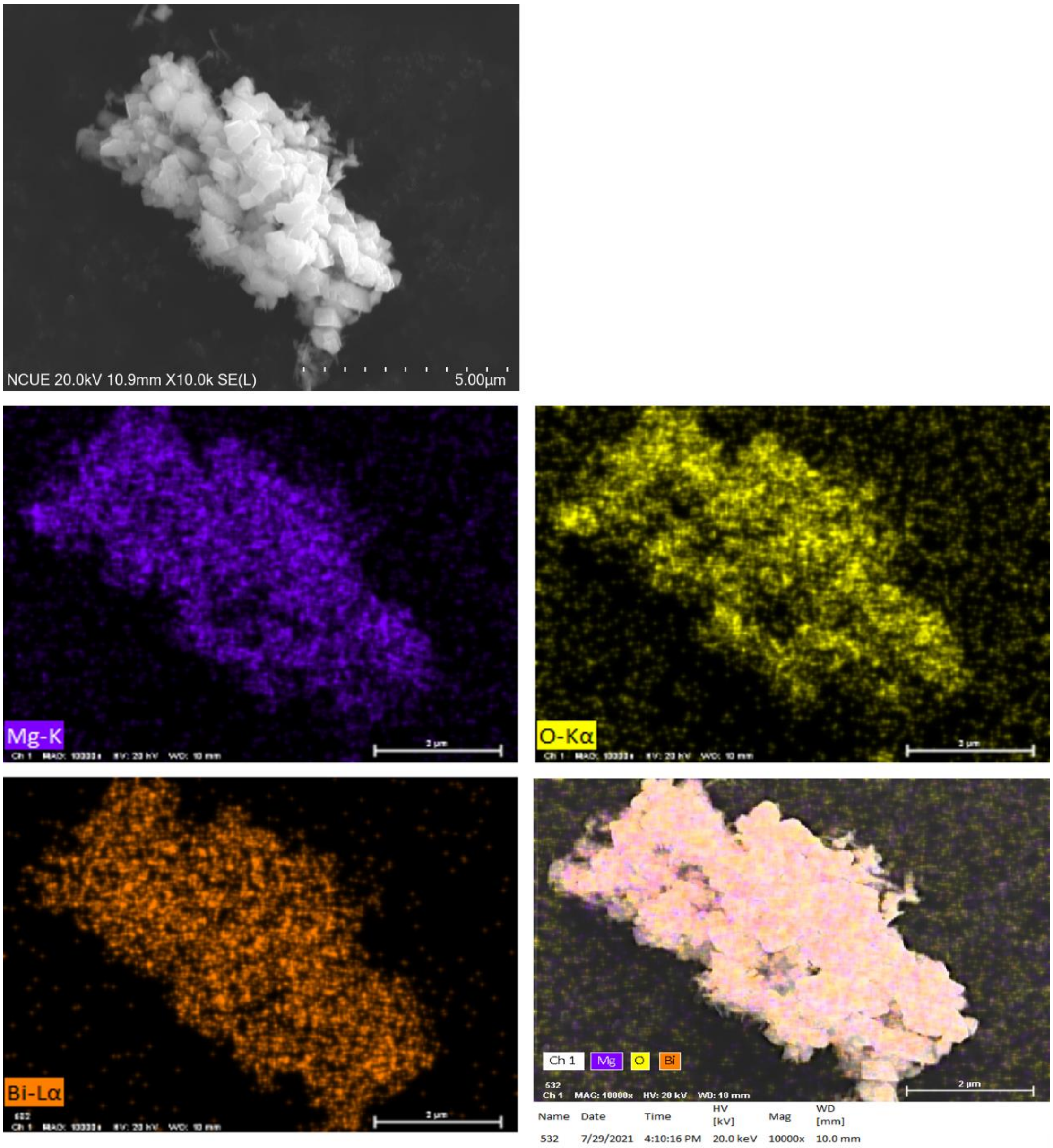

Figure S1. SEM image of $MgBi_2O_6$ and EDS-mapping of Mg, Bi, and O elements.

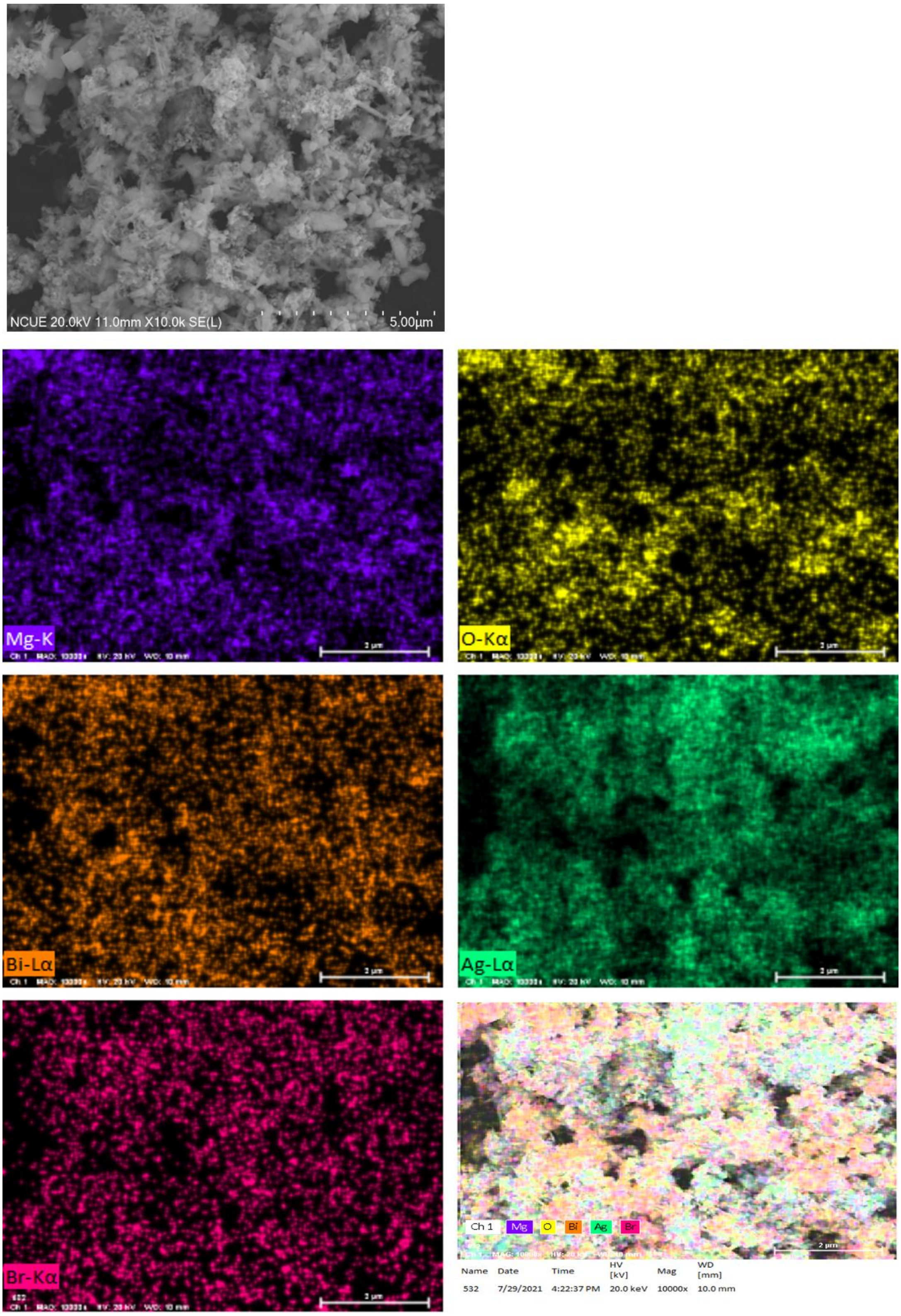

Figure S2. SEM image of $AgBr/MgBi_2O_6$ composites and EDS-mapping of Mg, Bi, and O elements

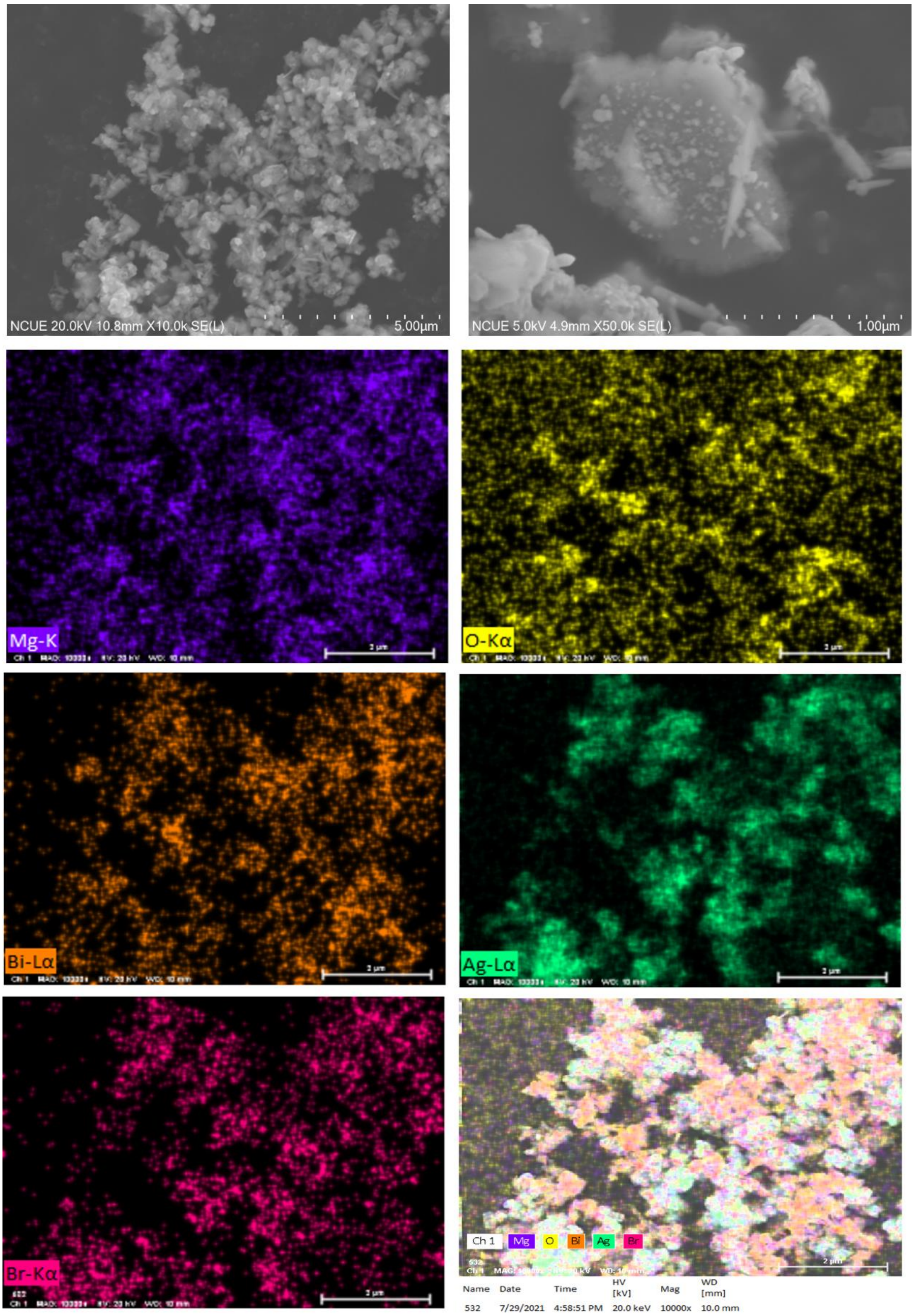

Figure S3. SEM images of $Ag/AgBr/MgBi_2O_6$ composites and EDS-mapping of Mg, Bi, and O elements

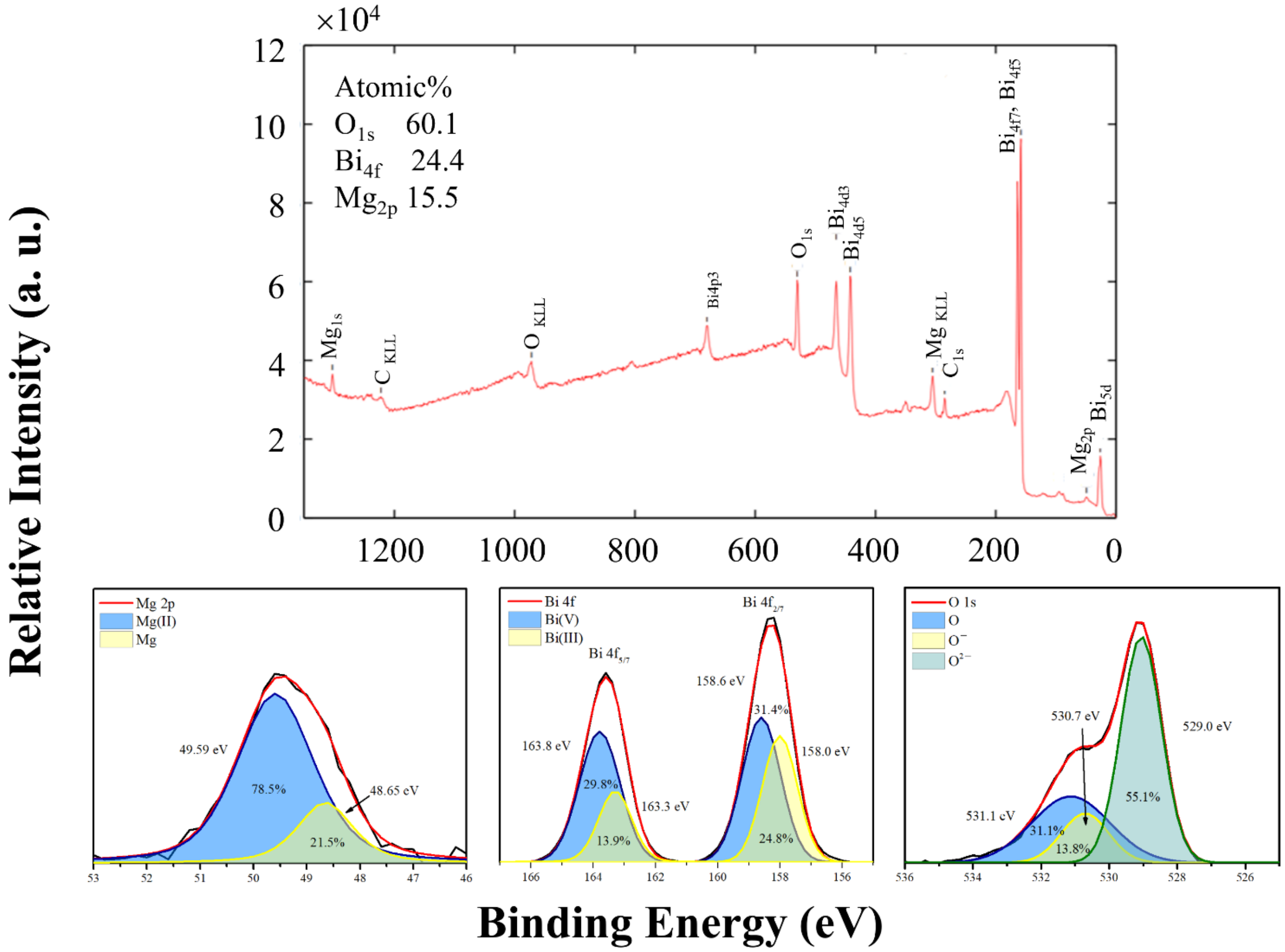

Figure S4. XPS analysis of $MgBi_2O_6$: full scan and high resolution of XPS for Mg, Bi, and O elements.

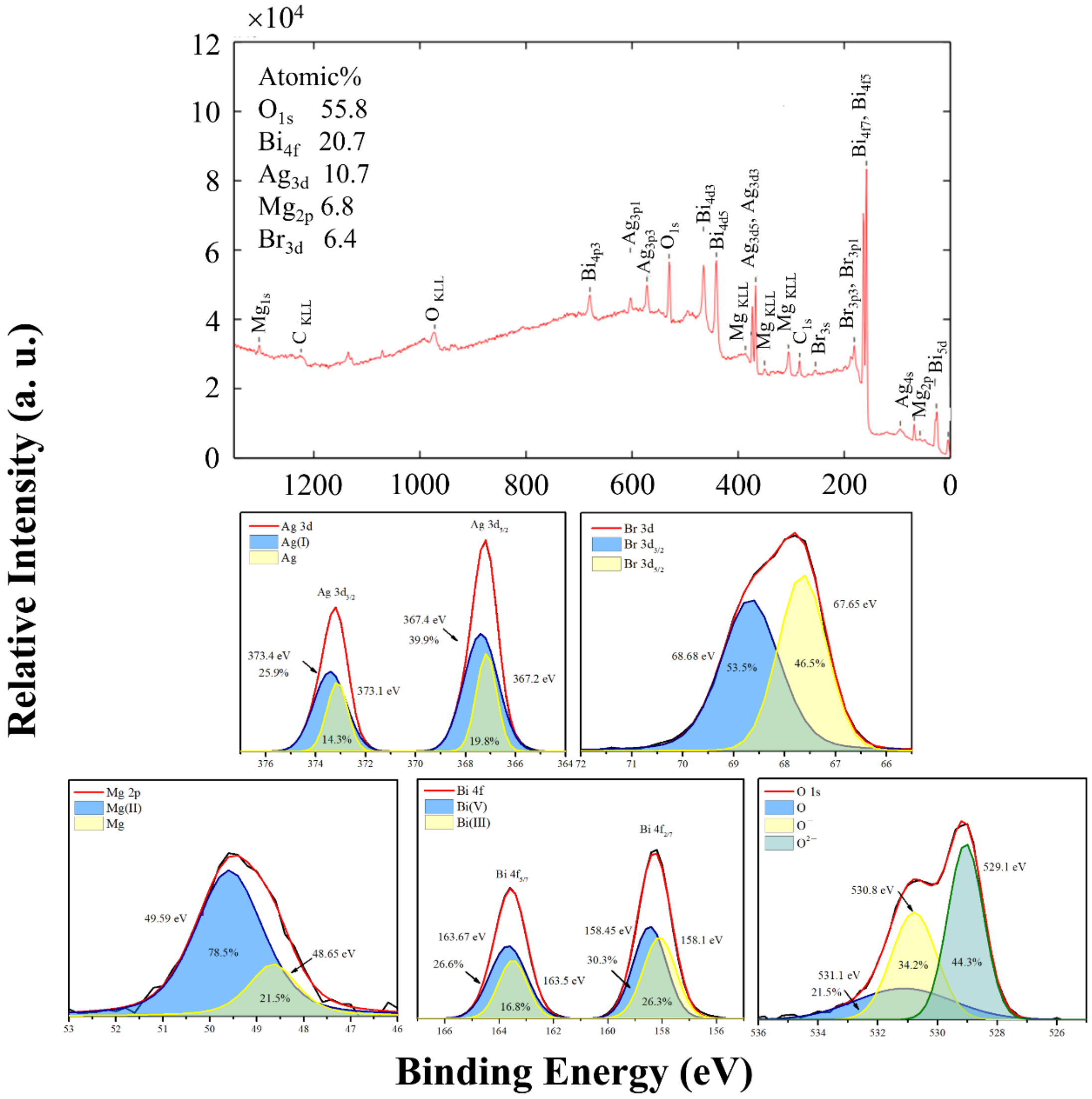

Figure S5. XPS analysis of $AgBr/MgBi_2O_6$ composites: full scan and high resolution of XPS for Ag, Br, Mg, Bi, and O elements.

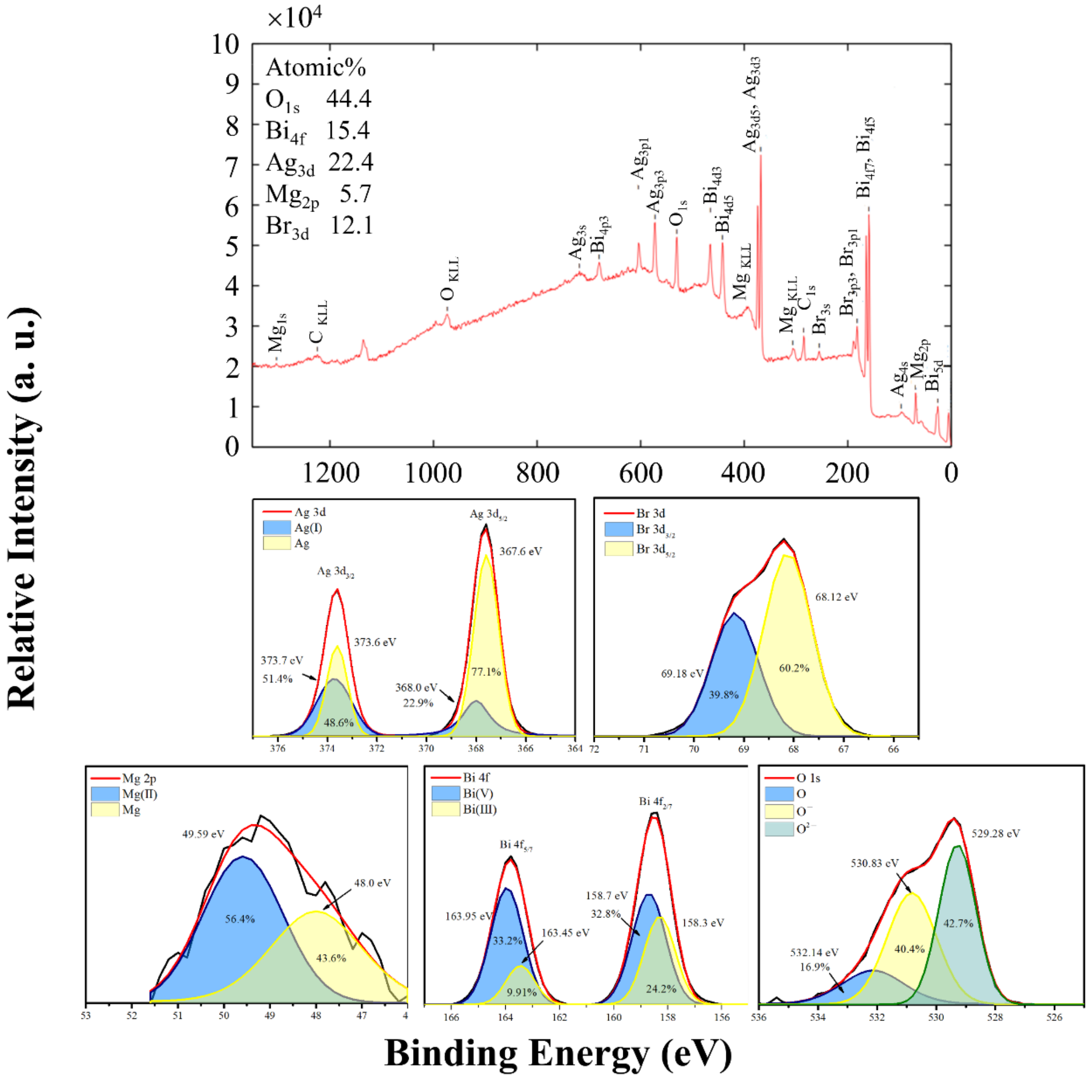

Figure S6. XPS analysis of $Ag/AgBr/MgBi_2O_6$ composites: full scan, and high resolution of XPS for Ag, Br, Mg, Bi, and O elements.

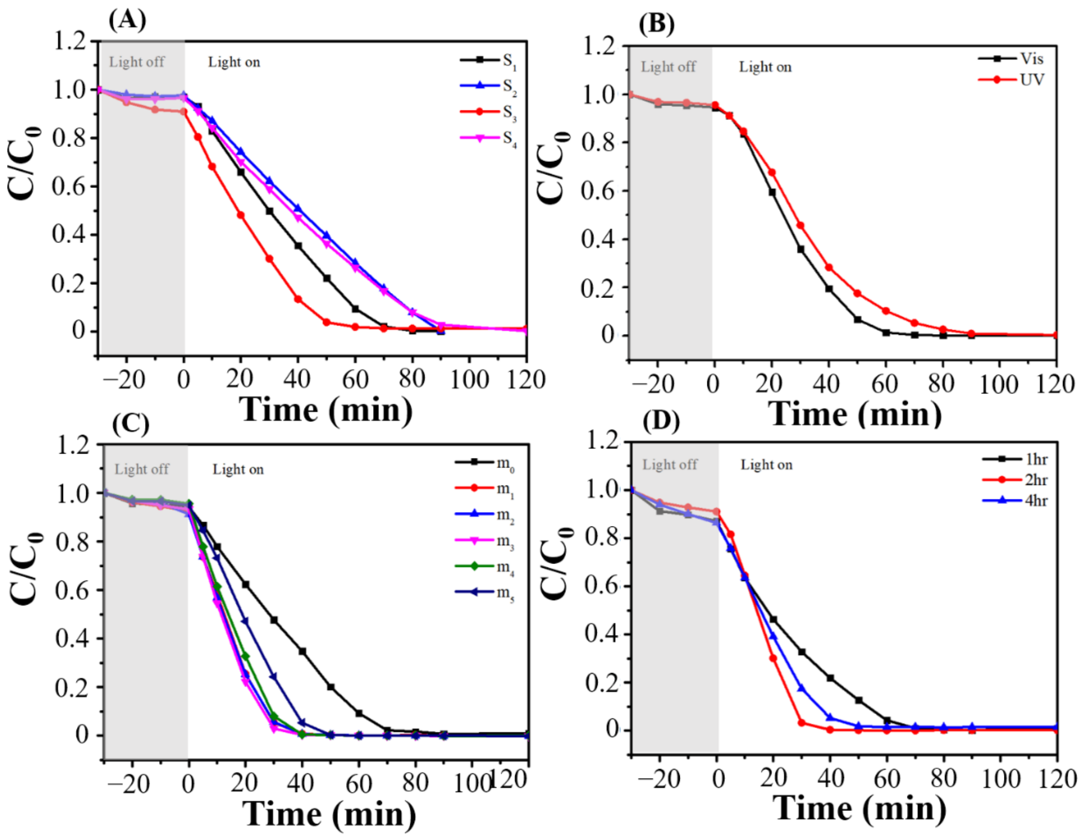

Figure S7. Optimum conditions of Ag/AgBr/$MgBi_2O_6$ composites: (A) different synthesis steps, (B) different light source irradiation, (C) different concentrations of $AgNO_3$, and (D) different irradiation times.
